# Health and Economic Outcomes of Addressing Encampments of Individuals Using Opioids

**DOI:** 10.1001/jamanetworkopen.2025.17095

**Published:** 2025-06-27

**Authors:** Hana Zwick, Ryan O’Dea, Joshua A. Barocas, Juliet Miko Flam-Ross, Avik Chatterjee, Alexander Y. Walley, Rebecca Arden Harris, Bruce R. Schackman, Laura F. White, Stavroula A. Chrysanthopoulou, Sabrina A. Assoumou, Sean M. Murphy, Jake R. Morgan, Dimitri Baptiste, Matthew Carroll, Benjamin Paul Linas

**Affiliations:** 1 Section of Infectious Diseases, Boston Medical Center, Boston, Massachusetts; 2 University of Colorado Anschutz Medical Campus, Aurora; 3 London School of Hygiene and Tropical Medicine, London, United Kingdom; 4 Boston University Chobanian and Avedisian School of Medicine, Boston, Massachusetts; 5 General Internal Medicine, Boston Medical Center, Boston, Massachusetts; 6 Department of Family Medicine, Perelman School of Medicine, University of Pennsylvania, Philadelphia; 7 Weill Cornell Medicine, New York, New York; 8 Boston University School of Public Health, Boston, Massachusetts; 9 Brown University School of Public Health, Providence, Rhode Island

## Abstract

**Importance:**

Many US communities face a crisis of people experiencing unsheltered homelessness often intertwined with opioid use. Jurisdictions seek policy options for managing unsanctioned encampments of this population, but their various outcomes are unclear.

**Objective:**

To evaluate policy options and their health and economic outcomes for an encampment of people experiencing homelessness and opioid use disorder (OUD).

**Design, Setting, and Participants:**

This decision analytical model study conducted a closed-cohort state-transition simulation using the Researching Effective Strategies to Prevent Opioid Death (RESPOND) model from October 2021 to October 2022. The study was based primarily on data from Massachusetts and simulated an urban encampment with a population experiencing homelessness and high-risk opioid use. Data analysis was performed from December 2022 to October 2024.

**Exposure:**

The following encampment management strategies were modeled: (1) status quo (no sweep); (2) sweep, a sudden disruption of all residents, followed by no additional resources; (3) housing with medication for opioid use disorder (MOUD) requirement; or (4) housing without MOUD requirement.

**Main Outcomes and Measures:**

The primary outcomes were overdose and all-cause mortality per 1000 person-years, weeks spent in housing and taking MOUD, and economic cost from a modified government payer perspective. Sensitivity analyses were conducted by varying uncertain parameters.

**Results:**

The simulated cohort included 400 adults (mean [SD] age, 48 [17] years; 232 males [58.0%]). Under the status quo strategy, there were 50.4 (95% uncertainty interval [UI], 48.9-52.2) deaths per 1000 person-years, 15.5 (95% UI, 14.0-17.2) deaths from overdose per 1000 person-years, and 2990 (95% UI, 2897-3081) person-weeks spent taking MOUD for a total cost of $6 583 000 (95% UI, $6 502 000-$6 660 000). A sweep strategy resulted in 53.1 (95% UI, 51.3-55.2) deaths per 1000 person-years, 16.4 (95% UI, 18.2-20.2) deaths from overdose per 1000 person-years, and 1694 (95% UI, 1625-1764) person-weeks spent taking MOUD at a total cost of $6 820 000 (95% UI, $6 736 000-$6 899 000). The housing with medication requirement strategy resulted in 51.2 (95% UI, 49.4-53.0) deaths per 1000 person-years, 16.3 (95% UI, 14.6-18.1) deaths from overdose per 1000 person-years, and 3050 (95% UI, 3025-3075) person-weeks spent taking MOUD and in housing, for a total cost of $7 264 000 (95% UI, $7 188 000-$7 336 000). A housing without MOUD requirement strategy resulted in 49.2 (95% UI, 47.6-51.1) deaths per 1000 person-years, 14.3 (95% UI, 12.7-16.2) deaths from overdose per 1000 person-years, and 5014 (95% UI, 4942-5085) person-weeks spent taking MOUD and 14 511 (95% UI, 14 461-14 562) person-weeks spent in housing, for a total cost of $8 822 000 (95% UI, $8 774 000-$8 868 000).

**Conclusions and Relevance:**

In this decision analytical model study of approaches to homeless encampments involving individuals with OUD, sweeps increased mortality and spending. Housing without MOUD requirement was the most costly strategy but saved more lives.

## Key Points

**Question:**

What are the health and economic outcomes of policy approaches to managing tent encampments of individuals experiencing homelessness and high-risk opioid use in Massachusetts?

**Findings:**

In this decision analytical model study of a simulated cohort of 400 adults, a city-sanctioned encampment sweep resulted in worse health outcomes than the status quo approach (leaving the encampment undisturbed), including more deaths, fewer person-weeks in treatment, and higher costs. The housing without medication for opioid use disorder (MOUD) requirement policy resulted in the lowest overall number of deaths and the highest number of person-weeks in treatment but required the highest cost.

**Meaning:**

The findings indicate that sweeps increase mortality and spending, whereas a policy of providing housing without MOUD requirement is costly but saves more lives.

## Teaser

This decision analytical model study evaluates the number of deaths and time receiving treatment associated with different strategies for responding to an urban encampment of adults experiencing homelessness and at high risk for opioid use disorder.

## Introduction

Many jurisdictions in the US are seeing an increase in people experiencing homelessness, a population with a high prevalence of opioid use disorder (OUD)^1,2^ who frequently live in unsanctioned encampments.^3^ While encampments can provide community and serve as access points for outreach services, local residents and government officials often express concerns regarding crime and adverse implications for businesses.^4,5^

One common response to encampments involves sweeps, in which government agencies disperse or arrest individuals living in these settings.^3^ Encampment sweeps have proven costly and harmful to the health of affected individuals^3,6,7,8^ and may divert resources from treatment services, harm reduction, or housing.^9^ Sweeps have been described as a form of structural violence, and evidence suggests that sweeps have a limited role in crime reduction.^5,10^ In response, some jurisdictions are piloting long-term housing for people living in encampments.^11,12^

Recognizing that housing programs may be concerned about ongoing substance use, one approach is to offer housing on the condition that OUD treatment is required. In this situation, an individual remains housed only if they continue using medication for OUD (MOUD). Housing First is an alternative approach that provides housing without requiring abstinence or MOUD engagement.^12^ Limited data exist regarding the effectiveness of either approach in improving the clinical outcomes of people with OUD. Some housing programs report decreased health care costs and increased housing retention,^12,13^ while others report either no appreciable changes to MOUD adherence or higher overdose rates.^14,15^

This study used a simulation model to compare encampment abatement strategies with the status quo of permitting encampments. A 2023 study using a different simulation model, found that encampment sweeps “worsen overdose and hospitalizations, decrease initiations of [MOUD], and contribute to deaths.”^3^ The current analysis extends those findings with an aim to evaluate policy options and their health and economic outcomes for an encampment of people experiencing homelessness and OUD.

## Methods

The Boston University Medical Campus and Boston Medical Center Institutional Review Board deemed this decision analytical model study exempt from review because it was not human participant research. This study followed the Consolidated Health Economic Evaluation Reporting Standards (CHEERS) reporting guideline.^16^ We used the Researching Effective Strategies to Prevent Opioid Death (RESPOND) simulation model, a cohort-based Markov model with a weekly time step to simulate the relapsing nature of OUD, overdose risk, and OUD treatment.^17^ We modeled the following strategies: (1) status quo (allowing the encampment to remain with current level of services); (2) performing a sweep that dismantles the encampment and disperses residents, followed by no additional resources; (3) housing contingent on continued treatment with MOUD; or (4) housing without MOUD requirement. We projected the outcomes from a modified government payer perspective that encompasses health care, carceral, housing, and sweep costs.

### RESPOND Model Structure

**Figure 1.**
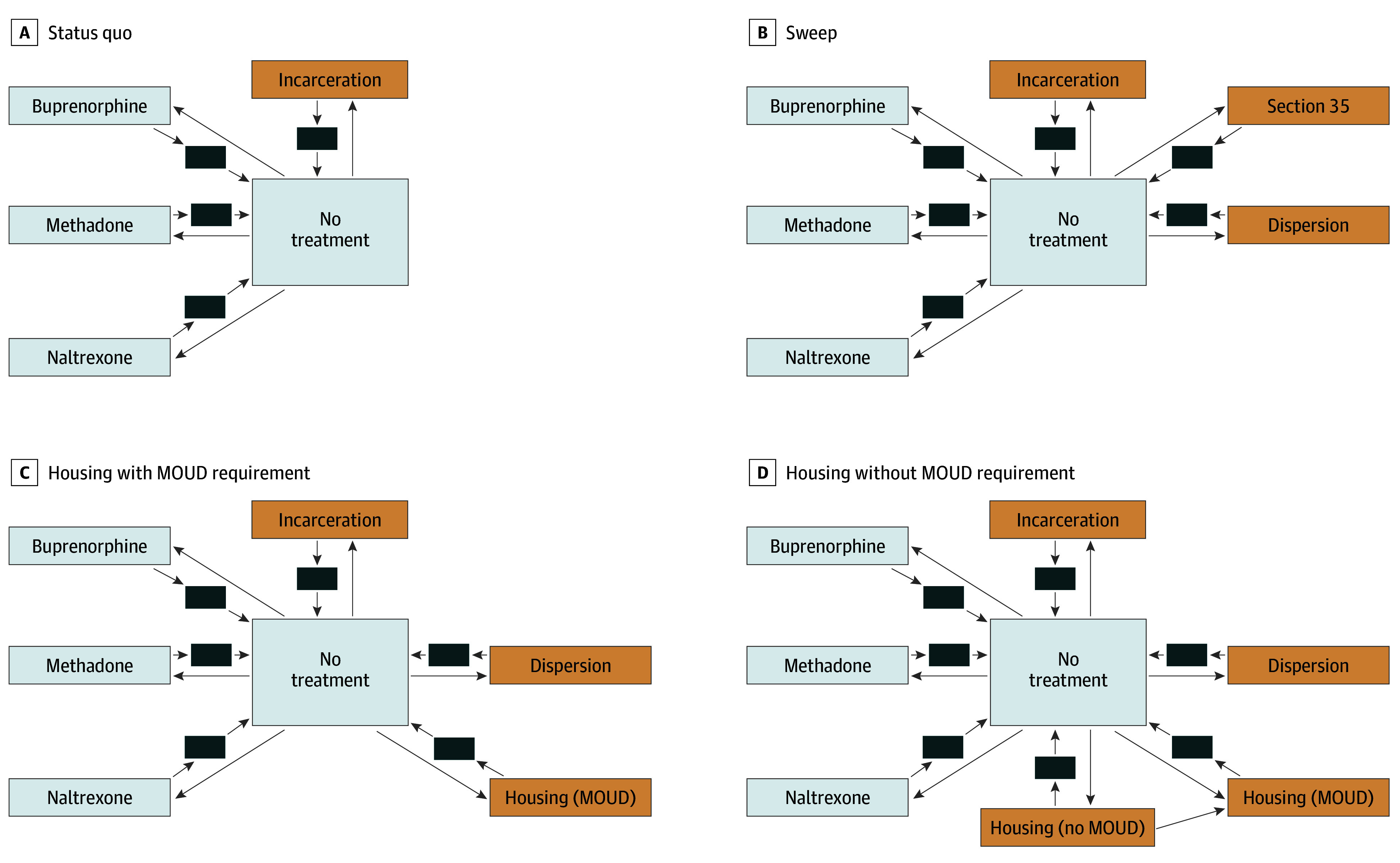
Researching Effective Strategies to Prevent Opioid Death (RESPOND) Model MOUD indicates medication for opioid use disorder.

The RESPOND model, described in detail in previous work and the eMethods in Supplement 1, is depicted in Figure 1 (eFigures 1-3 in Supplement 1).^18^ The model characterizes a series of transitions among 4 drug use states representing active vs nonactive and injection vs noninjection (eTables 1-2 in Supplement 1). Throughout the simulation, the population actively using opioids may seek MOUD (eTable 3 in Supplement 1). During treatment, movement occurs between active and nonactive drug use states with a bias toward nonactive (eTables 4-5 in Supplement 1). In every weekly time step, individuals engaged with MOUD may continue treatment, overdose and continue treatment, die from overdose or competing risks, or disengage from care (eTables 6-8 and 10-12 in Supplement 1). Each MOUD has an independent association with overdose beyond its association with substance use (eTable 9 in Supplement 1). The population has an incarceration risk that is higher when actively using drugs. During incarceration, we assumed the potential for ongoing drug use with risk of overdose mortality.^19^ After MOUD disengagement or release from correctional settings, individuals move through a posttreatment setting with an elevated overdose risk due to lowered opioid tolerance.^20^ Model parameters vary based on sex and age.

For the current analysis, we added 4 intervention states to the RESPOND model. The first intervention state is Section 35, a Massachusetts penal code that allows involuntary detainment and treatment for individuals deemed a risk to themselves or others. During a sweep, a proportion of individuals are sent to a Section 35 facility.^21^ After release, individuals either continue to take MOUD or transition through posttreatment to no treatment stages.^22^ Section 35 is a treatment option and differs from incarceration in length of stay and no possibility of overdose.

The second intervention state is dispersion, which many individuals enter after encampment disruption. A proportion of individuals engaged with MOUD lose access, experiencing an elevated risk of overdose due to loss of proximity to care. Additionally, with fewer overdose witnesses available to administer naloxone, the risk of death from opioid overdose is elevated.^23^

The third intervention state is housing with MOUD, wherein individuals may be given the option to avoid dispersion by moving into housing. We simulate MOUD in a supportive housing environment with the same effectiveness and costs as methadone in the community. We assume housing to be available before the sweep in the form of nontransitional single-site independent apartments or repurposed hotel rooms. Based on literature reviews,^24,25^ we assume that all individuals can receive onsite case management, mental health care, and MOUD services (eTable 13 in Supplement 1).

The fourth intervention state is housing without MOUD. The same assumptions about housing policies are applied here but without forced methadone engagement; however, individuals can initiate MOUD. Due to uncertainty in the literature,^25^ no changes to MOUD adherence or overdose rates are included in either housing setting.

### Data and Parameter Estimation

Key parameter values are listed in Table 1,^24,25,28,29,30,32,33,34,35,36,37,38,39,40,41,42,43,45,46^ with full model parameters in eTable 13 in Supplement 1. We used the medical literature^47,48,49,50^ to inform the natural history of OUD without treatment. Urine toxicological data from the National Institute on Drug Abuse Clinical Trials Network informed the bidirectional movement between active (relapse) and nonactive (remission) drug use while using MOUD (eTable 13 in Supplement 1).

**Table 1.** Project-Specific Model Parameters

Parameter	Baseline	Range evaluated in sensitivity analyses	Sources
Population demographic and epidemiologic characteristics			
Sex, proportion of total people at baseline			
Males	0.58	NA	Mass.gov,^26^ 2023; Opioid Policy Research Collaborative,^27^ 2023
Females	0.42	NA	
Age, mean (SD), y	48 (17)	NA	Mass.gov,^26^ 2023
Proportion receiving MOUD	0.20	NA	Mass.gov,^26^ 2023
Injection drug use, proportion of total people at baseline	0.25	NA	SAMHSA,^28^ 2023
Active drug use, proportion of total people at baseline	0.91	NA	CDC,^29^ 2018; Cedarbaum and Banta-Green,^30^ 2016
Mean (SD) standardized mortality rate for injection drug use, vs Massachusetts general population	8.09 (0.92)	7.3-8.9	Mass.gov,^26^ 2023; Roncarati et al,^31^ 2018
Mean (SD) standardized mortality rate for noninjection drug use, vs Massachusetts general population	3.27 (0.39)	2.9-3.6	Mass.gov,^26^ 2023; Roncarati et al,^31^ 2018
Transition to MOUD and housing, monthly rate per 1000 people			
Buprenorphine, mean (SD)	11.6 (5.89)	10.4-12.7	Mass.gov,^26^ 2023
Methadone, mean (SD)	3.7 (2.13)	3.3-4.0	Mass.gov,^26^ 2023
Injectable naltrexone, mean (SD)	2.4 (1.78)	2.2-2.6	Mass.gov,^26^ 2023
Housing with MOUD requirement	17.6	15.9-19.3	Mass.gov,^26^ 2023
Housing without MOUD requirement	17.1	15.4-18.8	Mass.gov,^26^ 2023
Housing with MOUD requirement from housing without MOUD requirement	21.65	0-43.30	Mass.gov,^26^ 2023
Length of stay			
Proportion of population that remains 6 mo after housing entry	0.98	0.44-1.00	MHSA,^32^ 2009; MHSA,^33^ 2017; Collins et al,^34^ 2013; Davidson et al,^35^ 2014; Stefancic and Tsemberis,^36^ 2007; Bean et al,^37^ 2013
Mean No. of days individuals remain in Section 35	20	1-90	Massachusetts Department of Public Health,^38^ 2019
Overdose, monthly rate per 1000 people, mean (SD)			
No treatment	7.5 (3.72)	6.5-8.5	Mass.gov,^26^ 2023
Buprenorphine	3.0 (1.52)	2.5-3.6	Mass.gov,^26^ 2023
Methadone	5.3 (3.23)	3.0-7.0	Mass.gov,^26^ 2023
Injectable naltrexone	5.4 (2.75)	2.6-7.3	Mass.gov,^26^ 2023
Corrections	3.8 (1.86)	3.3-4.3	Chatterjee et al,^39^ 2023^a^
Section 35 with mandatory treatment	0	0-4.3	No reference^b^
Dispersion	7.5 (3.72)	6.5-8.5	No reference^c^
Housing with MOUD requirement	5.3 (3.23)	3.0-7.0	No reference^d^
Housing without MOUD requirement	7.5 (3.72)	6.5-8.5	No reference^c^
Fatal overdoses, proportion of total overdoses			
All settings	0.28	0.25-0.31	Mass.gov,^26^ 2023; O’Driscoll et al,^40^ 2001
Pharmaceutical cost per wk, $			
Housing with MOUD requirement	4	NA	No reference^d^
Treatment utilization cost per wk, $			
Section 35	1477	NA	Swasey,^21^ 2019
Housing with MOUD requirement	Cost of methadone: 293; cost of housing: 122	NA	Cost of methadone: NIDA,^41^ 2023; cost of housing: MHSA,^32^ 2009; Latimer et al,^24^ 2020; Aubry et al,^25^ 2020; Jacob et al,^42^ 2022
Housing without MOUD requirement	293	NA	Cost of housing: MHSA,^32^ 2009; Latimer et al,^24^ 2020; Aubry et al,^25^ 2020; Jacob et al,^42^ 2022
Health care utilization cost, mean (SD) per wk, US $			
Housing with MOUD requirement	155 (41.45)	NA	MHSA,^32^ 2009; MHSA,^33^ 2017; Brown et al,^43^ 2015
Housing without MOUD requirement	155 (41.45)	NA	MHSA,^32^ 2009; MHSA,^33^ 2017; Brown et al,^43^ 2015
Sweep cost per person, $			
Total cost per person, $	275	156.15-393.74	NA
Component 1: cost of police per person, $	126	82-171	Bedford,^44^ 2022; HUD,^45^ 2020
Component 2: cost of garbage and sanitation per person, $	149	74-223	HUD,^45^ 2020; Swanson,^46^ 2021

Abbreviations: CDC, Centers for Disease Control and Prevention; HUD, Department of Housing and Urban Development; MHSA, Massachusetts Housing and Shelter Alliance; MOUD, medication for opioid use disorder; NA, not applicable; NIDA, National Institute on Drug Abuse; SAMHSA, Substance Abuse and Mental Health Services Administration.

^a^ Based on analysis by Chatterjee et al,^39^ we parameterized the overdose rate in correctional facilities to be half of the overdose rate in the no treatment stage. If the probability of overdose in the no treatment stage was 0.006 for a given demographic group, it would be 0.003 in correctional facilities for the same demographic group.

^b^ No reporting was found in the literature of overdoses within Section 35 facilities. Based on discussions with medical experts who work with individuals with substance use disorder, we assumed it was impossible to overdose within Section 35 facilities and conducted a sensitivity analysis to test that assumption.

^c^ In the dispersion and housing without MOUD states, individuals were not on treatment. We found no evidence to differentiate overdose rates in dispersion vs the encampment (no treatment) and therefore kept rates the same.

^d^ In the housing with MOUD state, individuals engaged with methadone. We therefore applied the overdose rate associated with methadone.

To inform treatment-seeking rates, we used the Massachusetts Public Health Data Warehouse (PHD), a longitudinally linked administrative records database that includes service encounter data from more than 16 statewide agencies.^26^ PHD data include overdose, overdose mortality, and treatment rates.

To tailor the simulation to individuals who were unhoused and used drugs, we increased nonoverdose mortality based on an analysis comparing mortality between unsheltered individuals experiencing homelessness and the general population of Massachusetts.^31^ In the simulation, nonoverdose mortality did not differ in housing settings due to a lack of data to inform such an outcome.

Remaining parameters were estimated through empirical calibration, yielding 6410 parameter vectors simulating PHD historical patterns (eTable 14 in Supplement 1). By running a strategy multiple times using different parameter vectors, we performed probabilistic sensitivity analyses and obtained possible ranges for simulated outcomes of interest. We sampled without replacement from the calibrated parameter vectors, obtaining 1000 vectors. For each of the 4 strategies, we completed 1000 runs using the same vectors.

#### Strategies

We modeled the following strategies: status quo, sweeps, housing with MOUD requirement, and housing without MOUD requirement. For the status quo strategy, the cohort progressed with no intervention. For the sweeps strategy, the cohort was subject to an encampment sweep. Based on expert opinion of collaborators from Boston-based public health institutions and data from an October 2021 sweep in Boston, we estimated that approximately 90% of individuals not receiving treatment would be dispersed and 10% would be committed to involuntary treatment under Section 35.^44^ Among individuals using MOUD at the time of the sweep, approximately 70% disengaged from care, 20% remained in treatment as usual, and 10% moved to Section 35.^44^ We experimented with those proportions in sensitivity analyses. For 1 month after the sweep, we increased overdose rates to reflect factors, including confiscation of life-saving interventions such as naloxone or fentanyl test strips, increased likelihood of using drugs alone, and general volatility.^3^

For the strategy of housing with MOUD requirement, at the time of the sweep, housing was offered to encampment residents, conditional on engagement with MOUD. We assumed that all individuals already using MOUD (20%) would be willing and able to be housed immediately, with subsequent housing uptake mirroring the general MOUD uptake. Those who were not previously receiving treatment were assumed to refuse treatment and moved to dispersion. We tested the impact of MOUD and housing acceptance rates in sensitivity analyses.

For the strategy of housing without MOUD requirement, at the time of the sweep, housing was offered to residents of the encampment with or without MOUD. We assumed that those who were using MOUD at the time of the sweep (20% of the total population) would maintain their engagement and accept housing. For those not using MOUD, housing without MOUD requirement policies allowed acceptance of housing without engaging with MOUD. We assumed 51% of the total population would accept housing without MOUD and the remaining 29% of the total population would refuse housing and move to dispersion.^51^ Individuals under this policy have the potential to engage with MOUD and move within the model from housing (no MOUD) to housing (MOUD). Based on data from Roundhouse (a transitional housing and addiction treatment center in Boston) and the literature, we assumed an MOUD uptake rate of 0.5% per week after individuals were housed.^11,52^

For each strategy, we projected overall mortality, fatal overdoses, person-weeks in MOUD and housing, and undiscounted costs. These were the primary outcomes.

#### Costs

We estimated costs of health care and treatment utilization using Medicare reimbursement schedules, which is considered best practice for estimating health care costs,^53^ and pharmaceutical costs from the Federal Supply Schedule Service (eTables 15-18 in Supplement 1). Overdose costs were obtained from the literature (eTables 19-20 in Supplement 1). We estimated the cost of conducting an encampment sweep from the literature at $275 per person per sweep (eTable 21 in Supplement 1). We conducted literature reviews to estimate the costs of providing housing, incarceration, and Section 35 facilities (eTables 13 and 21 in Supplement 1). The weekly cost of Section 35 ($1477 per person) was reported by the Massachusetts Department of Corrections. The weekly housing cost ($293 per person) was a mean of values across multiple sources in the literature based on housing implemented with a rent supplement of 30% of an individual’s income. The weekly cost of incarceration ($840) was based on data published in the *Federal Register*. All costs were reported in 2019 US dollars.

### Statistical Analysis

For all analyses, we used R, version 4.0.5 (R Project for Statistical Computing). Data analysis was performed from December 2022 to October 2024. The analysis began with the cohort living in the encampment. We ran the simulation from October 2021 to October 2022, choosing a short time horizon because a longer time horizon might not reflect the perspective of decision-makers grappling with these issues.

We performed probabilistic sensitivity analyses by running each strategy 1000 times, sampling from the probability distribution around key model parameters and aggregating the results (eTable 22 in Supplement 1). We also performed a series of deterministic sensitivity analyses (DSAs), varying key parameters one at a time to the upper and lower bounds of their feasible ranges (eTable 22 in Supplement 1) as defined by expert opinion and maximum values. Notable DSAs included proportions of individuals who transitioned between drug use states at the time of the sweep, transitions from active to nonactive substance use, movements into each setting (MOUD and housing) at the time of and after the sweep, overdose rates, and proportion of overdoses that were fatal.

## Results

### Strategies

The simulated cohort included 400 adults, of whom 232 were males (58.0%) and 168 were females (42.0%) with a mean (SD) age of 48 (17) years.^26,27,54^ Results are presented in Table 2 as the median of 1000 runs. Total deaths and fatal overdoses are presented per 1000 person-years, time is presented in person-weeks, and costs are in US dollars. Uncertainty intervals (95% UIs) reflect the 2.5th and 97.5th percentile of the results of the 1000 runs but require careful interpretation due to the association of parameters across scenarios (eFigure 4 and eTable 15 in Supplement 1).

**Table 2.** Results of 1000 Runs

Variable	Median (95% UIs)			
	Status quo	Sweeps	Housing with MOUD requirement	Housing without MOUD requirement
Fatal overdoses per 1000 person-years	15.5 (14.0-17.2)	16.4 (18.2-20.2)	16.3 (14.6-18.1)	14.3 (12.7-16.2)
Nonoverdose fatalities per 1000 person-years	34.9 (31.7-38.2)	36.7 (31.1-37.0)	34.9 (31.3-38.4)	34.9 (33.3-38.4)
Total fatalities per 1000 person-years	50.4 (48.9-52.2)	53.1 (51.3-55.2)	51.2 (49.4-53.0)	49.2 (47.6-51.1)
Time spent in housing, person-weeks	0	0	3050 (3025-3075)	14 511 (14 461-14 562)
Time spent taking MOUD, person-weeks	2990 (2897-3081)	1694 (1625-1764)	3050 (3025-3075)	5014 (4942-5085)
Total cost, US $	6 583 000 (6 502 000-6 660 000)	6 820 000 (6 736 000-6 899 000)	7 264 000 (7 188 000-7 336 000)	8 822 000 (8 774 000-8 868 000)
Health care utilization cost, US $	6 135 000 (6 065 701-6 203 253)	6 202 000 (6 127 000-6 275 000)	5 895 000 (5 825 000-5 956 000)	3 849 000 (3 817 000-3 875 000)
Pharmaceutical cost, US $	144 000 (140 000-149 000)	79 000 (75 000-82 000)	13 000 (13 000-13 000)	22 000 (21 000-22 000)
Treatment utilization cost, US $	233 000 (226 000-240 000)	357 000 (352 000-362 000)	384 000 (379 000-389 000)	627 000 (618 000-636 000)
Overdose cost, US $	71 000 (63 000-78 000)	74 000 (67 000-82 000)	73 000 (66 000-82 000)	65 000 (58 000-73 000)
Sweep cost, US $	0	110 000 (NA)	0	0
Housing cost, US $	0	0	899 000 (886 000-912 000)	4 259 000 (4 195 000-4 323 000)

Abbreviations: MOUD, medication for opioid use disorder; NA, not applicable.

**Figure 2.**
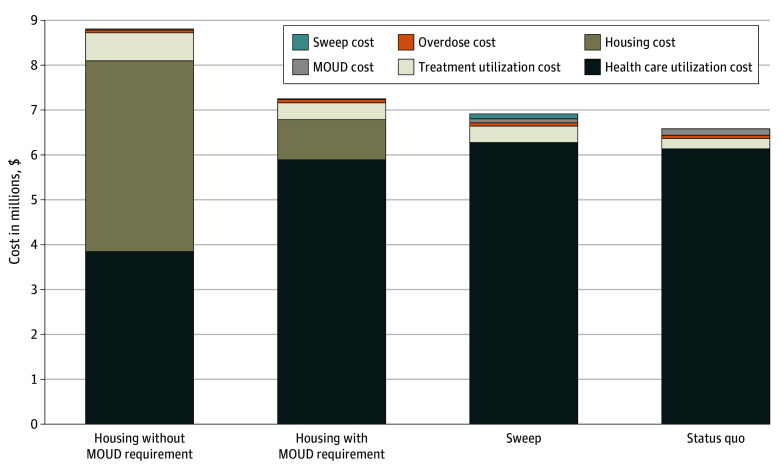
Cost of Strategies by Category at 1-Year Time Horizon MOUD indicates medication for opioid use disorder.

Under the status quo strategy, there were 50.4 (95% UI, 48.9-52.2) deaths per 1000 person-years, 15.5 (95% UI, 14.0-17.2) deaths per 1000 person-years due to overdose, and 2990 (95% UI, 2897-3081) person-weeks spent taking MOUD, with a total cost of $6 583 000 (95% UI, $6 502 000-$6 660 000). Conducting the sweep strategy led to 53.1 (95% UI, 51.3-55.2) deaths per 1000 person-years, 16.4 (95% UI, 18.2-20.2) deaths per 1000 person-years from overdose, and 1694 (95% UI, 1625-1764) person-weeks spent taking MOUD. The sweep strategy cost $6 820 000 (95% UI, $6 736 000-$6 899 000), $237 000 (3.6%) more than the cost of the status quo strategy. This amount included $109 000 from direct implementation of the sweep, and the remainder consisted of increased overdoses and health care utilization (Figure 2).

We compared the housing strategies first to the sweep strategy. The housing with MOUD requirement strategy resulted in 51.2 (95% UI, 49.4-53.0) deaths per 1000 person-years, 16.3 (95% UI, 14.6-18.1) deaths per 1000 person-years from overdose, and 3050 (95% UI, 3025-3075) person-weeks spent taking MOUD, for a total cost of $7 264 000 (95% UI, $7 188 000-$7 336 000). There were 3050 (95% UI, 3025-3075) person-weeks spent in housing. The nature of costs categorically shifted away from treating complications of drug use and toward providing OUD treatment and housing (Figure 2).

When individuals were offered housing without MOUD requirement, there were 49.2 (95% UI, 47.6-51.1) total deaths per 1000 person-years (vs sweep), 14.3 (95% UI, 12.7-16.2) deaths per 1000 person-years from overdose, and 5014 (95% UI, 4942-5085) person-weeks spent taking MOUD, for a total cost of $8 822 000 (95% UI, $8 774 000-$8 868 000). There were 14 511 (95% UI, 14 461-14 562) person-weeks spent in housing, a large increase compared with both sweep and housing with MOUD requirement strategies. Again, the nature of costs shifted away from managing complications of drug use and toward providing OUD treatment and housing (Figure 2).

When we compared the clinical outcomes of the housing strategies with the status quo strategy, housing with MOUD requirement had worse public health and economic outcomes. Total deaths increased by 0.2 per 1000 person-years, overdose deaths increased by 0.3 per 1000 person-years, and total costs increased by $681 000. The housing without MOUD requirement (compared with the status quo) had improved results in all 3 health outcomes considered.

### Sensitivity Analyses

In DSAs, there was no scenario in which the sweep policy had the best outcomes. One important dynamic for the impact of housing strategies was the rate of housing acceptance. In the housing with MOUD requirement strategy, total deaths exceeded the rates in the status quo strategy when fewer than 25.0% of individuals accepted MOUD to secure housing (eFigure 5 in Supplement 1).

**Figure 3.**
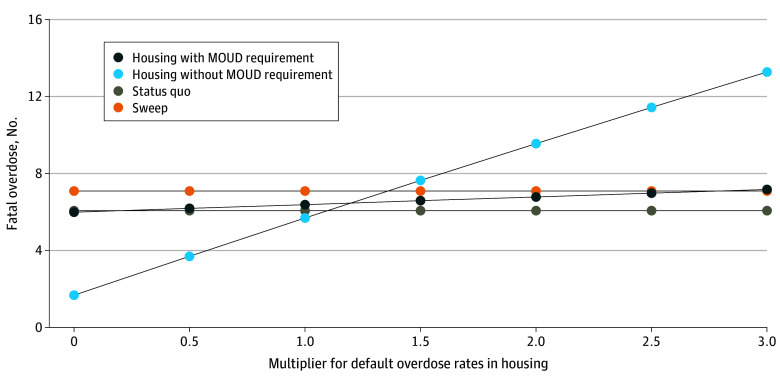
Fatal Overdoses by Strategy and Housing Overdose Multiplier Applied to Both Housing Strategies MOUD indicates medication for opioid use disorder.

Outcomes were sensitive to overdose rates. The overdose rate in housing would need to increase from 6.40 per 1000 people per month in the encampment to 17.28 for the housing with MOUD requirement policy to result in more overdoses than the sweep strategy and to 8.96 for the housing without MOUD requirement strategy to result in more fatal overdoses than the sweep policy. Figure 3 shows the number of fatal overdoses in each strategy based on the relative increase in overdose in housing.

We conducted further DSAs, including maintaining higher MOUD engagement during the sweep, moving different proportions of people to Section 35, assuming no MOUD uptake in the housing without MOUD requirement strategy, and increasing overdose rates in housing. In none of those scenarios did the sweep strategy provide better outcomes than either housing strategy. All sensitivity analysis results are provided in eTable 23 in Supplement 1.

## Discussion

Encampment sweeps are a common response to unsanctioned homeless encampments, often implemented without provision of long-term supportive housing.^44,51^ Our findings shed light on the outcomes associated with different strategies for addressing homelessness among individuals with OUD, indicating that encampment sweeps incur higher costs and more fatalities than leaving the encampment undisturbed or providing supportive housing.

This study provides a projection of the full cost of an encampment sweep, which is difficult to empirically observe. While a US city might budget $109 000 for police, sanitation, and other services, that does not include all monetary costs. Over the 12-month period following a disruption, the total cost of conducting a sweep was approximately $237 000, more than twice the cost of resources consumed to conduct the sweep itself.

Additionally, the findings demonstrate that the approach a jurisdiction takes to offer housing directly affects mortality and cost. An offer of housing contingent on MOUD engagement can be associated with increased mortality compared with a counterfactual status quo. While making housing conditional on OUD treatment is intuitively appealing and may represent a comfortable middle ground to policymakers who are seeking to balance competing agendas, this current analysis demonstrates that, if the uptake of MOUD-linked housing falls below approximately 25.0%, the strategy continues to result in increased mortality and cost.

Plausible scenarios where people in housing may use drugs in isolation may be associated with more unwitnessed overdoses and deaths than in encampments.^55,56^ Our findings offer reassurance that, even with some increase in overdose risk, the overall mortality in housing is unlikely to rise above that of an encampment. This finding is critical in considering housing in the context of a volatile drug supply, which can play a role in rapidly changing overdose mortality.

### Limitations

This study has multiple limitations. The RESPOND simulation model excluded costs from the perspective of the surrounding community and the individuals in the encampment. This trade-off is complex, although the losses to the community (business revenue and, potentially, quality of life) are categorically different from the losses of life in the encampment. While there is no correct assessment of this trade-off, the losses from sweeps are undeniably large.

We simulated care delivery in Massachusetts, and thus projections may not generalize to other states. Nonetheless, nearly every major US city hosts many unsheltered individuals; thus, comparable results may be expected.^3^ Data are unavailable regarding impacts by subgroups, such as race and ethnicity, sex, or opioid use status, although sensitivity analyses may allow for some high-level conclusions to be drawn. Data are also limited regarding specific outcomes of recent sweeps and housing; therefore, we relied on sources, such as news articles, to estimate some parameters. However, the core findings and messages of this study were robust across broad parameter value ranges.

The RESPOND model did not simulate capacity limitations in any intervention. We also assumed that housing was move-in ready, without upfront development costs. Our analysis was limited to placement in long-term, single-site, apartment housing. In reality, multiple types of housing may be considered, changing costs as well as uptake and retention of housing. These limitations provide opportunities for future research to provide insight on outcomes and costs of sweeps and housing over multiple years.

## Conclusions

This decision analytical modeling study of approaches to homeless encampments, including sweeps with and without alternative housing options, found that for unsheltered individuals using opioids, sweeps led to preventable deaths and diverted essential funds from overdose crisis interventions. Supportive housing initiatives yielded better health outcomes but required substantial resources. The results of the current study could inform policymaking by identifying the estimated economic cost and serious adverse impacts of sweeps, showing that sweeps alone are an ineffective way of mitigating the US homelessness crisis.

## Notes

Supplement 1.
eMethods.
**eFigure 1.** Core Simulation **eFigure 2.** RESPOND’s Care Delivery Module With Project-Specific Strategies Represented **eTable 1.** Initializing Cohort Parameters **eTable 2.** No-Treatment and Post-Treatment: OUD Transition Parameters **eTable 3.** Weekly Transition Probabilities From No-Treatment to Treatment **eTable 4.** Block Initiation Effects Parameters: Weekly Transition Probabilities Modeling Movement Between OUD States When Movement Between Treatment States Occurs **eFigure 3.** Core Simulation Within OUD Treatment Episodes (Blocks) **eTable 5.** Transition Probabilities Between Active and Non-Active Opioid Use While Engaged With Treatment **eTable 6.** Weekly Transition Probabilities From Treatment *T* to Post-Treatment **eTable 7.** Empirically Observed Opioid Overdoses in MA **eTable 8.** Opioid Overdose Rates by Age, Sex, Year, and Type of OUD **eTable 9.** Multipliers of Overdose Rates by Treatment *T* **eTable 10.** Probabilities of Fatal Overdose **eTable 11.** Overdose Excluded Weekly Death Probabilities Derived From Lifetables **eTable 12.** Standardized Mortality Rates (SMRs) **eTable 13.** Model Parameters **eTable 14.** List of Calibration Parameters of RESPOND **eTable 15.** Pharmaceutical Costs **eTable 16.** Cost of Naltrexone Treatment for Opioid Use Disorder **eTable 17.** Cost of Buprenorphine Treatment for Opioid Use Disorder **eTable 18.** Cost of Methadone Treatment for Opioid Use Disorder **eTable 19.** Cost of Nonfatal Overdose **eTable 20.** Cost of Fatal Overdose **eTable 21.** Cost of Interventions **eFigure 4.** Fatal Overdoses by Strategy and Parameter Set **eTable 22.** Sensitivity Analyses Upper and Lower Bounds **eTable 23.** Sensitivity Analysis Results **eFigure 5.** Projected Deaths by Strategy and Proportion of Population Accepting Housing With MOUD at the Time of the Sweep
eReferences

Supplement 2.
Data Sharing Statement
