# Supplementary material for: Health and Economic Outcomes of Addressing Encampments of Individuals Using Opioids
Source: JAMA Netw Open. 2025 Jun 27;8(6):e2517095. doi: 10.1001/jamanetworkopen.2025.17095 (PMC12205401; doi:10.1001/jamanetworkopen.2025.17095)
Supplement: Supplement 1. — eMethods. eFigure 1. Core Simulation eFigure 2. RESPOND’s Care Delivery Module With Project-Specific Strategies Represented eTable 1. Initializing Cohort Parameters eTable 2. No-Treatment and Post-Treatment: OUD Transition Parameters eTable 3. Weekly Transition Probabilities From No-Treatment to Treatment eTable 4. Block Initiation Effects Parameters: Weekly Transition Probabilities Modeling Movement Between OUD States When Movement Between Treatment States Occurs eFigure 3. Core Simulation Within OUD Treatment Episodes (Blocks) eTable 5. Transition Probabilities Between Active and Non-Active Opioid Use While Engaged With Treatment eTable 6. Weekly Transition Probabilities From Treatment T to Post-Treatment eTable 7. Empirically Observed Opioid Overdoses in MA eTable 8. Opioid Overdose Rates by Age, Sex, Year, and Type of OUD eTable 9. Multipliers of Overdose Rates by Treatment T eTable 10. Probabilities of Fatal Overdose eTable 11. Overdose Excluded Weekly Death Probabilities Derived From Lifetables eTable 12. Standardized Mortality Rates (SMRs) eTable 13. Model Parameters eTable 14. List of Calibration Parameters of RESPOND eTable 15. Pharmaceutical Costs eTable 16. Cost of Naltrexone Treatment for Opioid Use Disorder eTable 17. Cost of Buprenorphine Treatment for Opioid Use Disorder eTable 18. Cost of Methadone Treatment for Opioid Use Disorder eTable 19. Cost of Nonfatal Overdose eTable 20. Cost of Fatal Overdose eTable 21. Cost of Interventions eFigure 4. Fatal Overdoses by Strategy and Parameter Set eTable 22. Sensitivity Analyses Upper and Lower Bounds eTable 23. Sensitivity Analysis Results eFigure 5. Projected Deaths by Strategy and Proportion of Population Accepting Housing With MOUD at the Time of the Sweep eReferences [file jamanetwopen-e2517095-s001.pdf]

Supplemental Online Content

Zwick H, O'Dea R, Barocas JA, et al. Health and economic outcomes of addressing encampments of individuals using opioids. *JAMA Netw Open*. 2025;8(6):e2517095.
doi:10.1001/jamanetworkopen.2025.17095

eMethods

eFigure 1. Core Simulation

eFigure 2. RESPOND's Care Delivery Module With Project-Specific Strategies Represented

eTable 1. Initializing Cohort Parameters

eTable 2. No-Treatment and Post-Treatment: OUD Transition Parameters

eTable 3. Weekly Transition Probabilities From No-Treatment to Treatment

eTable 4. Block Initiation Effects Parameters: Weekly Transition Probabilities Modeling Movement Between OUD States When Movement Between Treatment States Occurs

eFigure 3. Core Simulation Within OUD Treatment Episodes (Blocks)

eTable 5. Transition Probabilities Between Active and Non-Active Opioid Use While Engaged With Treatment

eTable 6. Weekly Transition Probabilities From Treatment *T* to Post-Treatment

eTable 7. Empirically Observed Opioid Overdoses in MA

eTable 8. Opioid Overdose Rates by Age, Sex, Year, and Type of OUD

eTable 9. Multipliers of Overdose Rates by Treatment *T*

eTable 10. Probabilities of Fatal Overdose

eTable 11. Overdose Excluded Weekly Death Probabilities Derived From Lifetables

eTable 12. Standardized Mortality Rates (SMRs)

eTable 13. Model Parameters

eTable 14. List of Calibration Parameters of RESPOND

eTable 15. Pharmaceutical Costs

eTable 16. Cost of Naltrexone Treatment for Opioid Use Disorder

eTable 17. Cost of Buprenorphine Treatment for Opioid Use Disorder

© 2025 Zwick H et al. *JAMA Network Open*

eTable 18. Cost of Methadone Treatment for Opioid Use Disorder

eTable 19. Cost of Nonfatal Overdose

eTable 20. Cost of Fatal Overdose

eTable 21. Cost of Interventions

eFigure 4. Fatal Overdoses by Strategy and Parameter Set

eTable 22. Sensitivity Analyses Upper and Lower Bounds

eTable 23. Sensitivity Analysis Results

eFigure 5. Projected Deaths by Strategy and Proportion of Population Accepting Housing With MOUD at the Time of the Sweep

eReferences

This supplemental material has been provided by the authors to give readers additional information about their work.

eMethods

A. Introduction

The growing prevalence of Opioid Use Disorder (OUD) has resulted in an increase in opioid overdoses in the United States. Drug overdose is the leading cause of premature death among Americans under the age of 50 and has increased by more than 2.5 times between 1999 and 2015. Although evidence-based treatments are available for treating OUD, these treatments are under-utilized, thus the impact of opioids on the United States' population persists.

Researchers and policy makers have made efforts to create feasible action plans for reducing the prevalence of OUD. Unfortunately, most policy makers do not have the evidence needed for informing and implementing system-level change. System-level thinking investigates how systems operate and how they can be modified to produce desired outcomes. At this time, data on system-level interventions for OUD are limited and inconsistent.

In an effort to fill the knowledge gap, simulation modeling can be used to integrate data from multiple sources to translate outcomes from clinical studies to policy-relevant data about population health and cost. By simulating state-level behaviors and practices related to OUD, we can project and evaluate the impact of relevant interventions and policies on public health outcomes and costs, hence informing practice and policy decisions to combat OUD.

The **R**esearching **E**ffective **S**trategies to **P**revent **O**pioid **D**eath (RESPOND) model is a state-transition, cohort-based model that simulates populations with high-risk opioid use in Massachusetts, including the natural history of OUD, movement on and from opioid treatment, and overdose. The model provides outputs and projections that decision-makers can use to evaluate and modify care delivery systems to match their local epidemics and available resources.

Model inputs and parameters are adaptable to users' needs, namely, to represent heterogeneous populations, different dynamics of the drug overdose epidemic, and the effectiveness of intervention strategies in the prevention of opioid-related harms. The user, for example, can customize among other things, the demographics, time in each cycle, transition probabilities between health statuses and treatment states, and the number of health states included in the model to represent different structures and disease dynamics of the underlying populations.

B. Model Structure

B.1 Overview

RESPOND is a state-transition, cohort-based model that simulates the population living within a jurisdiction and who have high-risk opioid use.[1,2] Typically, RESPOND simulates the population of a state, but it can also simulate a smaller area, such as a town or rural community, depending on the model parameter values. The model employs a Markov process with a weekly cycle length to accurately reflect population dynamics, clinical progression, and treatment of OUD.

The model structure comprises four main components: 1) population dynamics, 2) natural history of OUD, 3) care delivery, and 4) mortality.

The population dynamics modules simulate the epidemiology and demography of the opioid epidemic. The user can create either an open or a closed cohort simulation. In an open cohort simulation, new population "arrives" to the simulation in every time step such that the total population in the model always reflects the size of the total population with OUD living in that jurisdiction. The arrival rate represents both the development of new OUD and migration into the state among those with existing opioid use. In a closed cohort, no cohort members enter the simulation and the size of the population in the simulation dwindles over time as cohort members die.

The core simulation (*eFigure 1*) of the RESPOND model involves the simulation of the natural history of OUD as a relapsing and remitting disease over a lifetime. RESPOND simulates OUD as a series of transitions between four health states of opioid use: 1) active, non-injection, 2) non-active, non-injection, 3) active injection, and 4) non-active, injection opioid use. In each time-step of the simulation, population fractions move between opioid use states. The definitions of "active" and "injection" opioid use can vary (but must be pre-specified) depending on the users' needs and available information. In the RESPOND Massachusetts base case, "active" opioid use is defined as any reported use in the previous seven days. "Injection" opioid use reflects any injection in the preceding seven days (a person who is both injecting and using oral opioids would be categorized as "injection" in RESPOND).

© 2025 Zwick H et al. *JAMA Network Open*

The care delivery module (*Figure 2)* of RESPOND simulates OUD treatment and includes four treatment types: 1) outpatient buprenorphine (Bup), 2) outpatient injectable naltrexone (Ntx), 3) outpatient methadone (Mmt) maintenance, and 4) inpatient acute drug detoxification (detox). The model is adaptable to additional intervention types to better reflect local conditions and evolutions in the treatment field. In general, treatment episodes tend to decrease movement into active drug use, increase movement into non-active drug use, and have an independent effect on overdose rates conditional on active drug use. When population disengages from a treatment and is lost to follow-up, those people enter a corresponding "post-treatment state". The post-treatment state is a fixed interval during which relapse to active drug use is high, tolerance to opioids is lower than before treatment, and the risk of drug overdose among those actively using opioids is higher than it is in the no treatment state. The post-treatment state represents the period of vulnerability and excess overdose observed in real-world settings among patients who have recently relapsed to opioid use after a period of sustained abstinence.

The mortality module simulates both drug-related and competing risks deaths. RESPOND simulates overdose mortality by first simulating overdose incidence as a function of age and type of drug use (injection vs. non-injection use). Next, the model simulates a probability of death conditional on having had an opioid overdose. The model simulates competing causes of death using standardized mortality ratios that are a function of age, sex, and type of opioid use (injection vs. non-injection).

The primary model outputs are: 1) All-cause mortality, 2) Overdose mortality, and 3) Number of people on treatment.

The simulation process is as follows: At simulation start, the model initiates a cohort of people currently living with OUD in the jurisdiction of interest. Based on data from that jurisdiction, the model assigns the current population to a drug use state, as well as a treatment block, such that the simulated population, including the prevalence of OUD treatment, reflects the status quo. Moving forward through simulated time, the sequence of simulation steps is: 1) aging of the population, 2) arrival of new population, 3) transition between OUD drug use states, 4) transitions into and out of treatment, 5) overdose, and 6) death. At the end of this sequence of processes, the model advances simulated time by one cycle (week) and repeats the process. The simulation continues until a time horizon assigned by the user.

Full model schematics are available at https://www.syndemicslab.org/respond.

Following sections of this document provide details of model parameters for a specific use case of the model that simulates the OUD population of the state of Massachusetts from the end of year 2012-2015 with an open cohort.

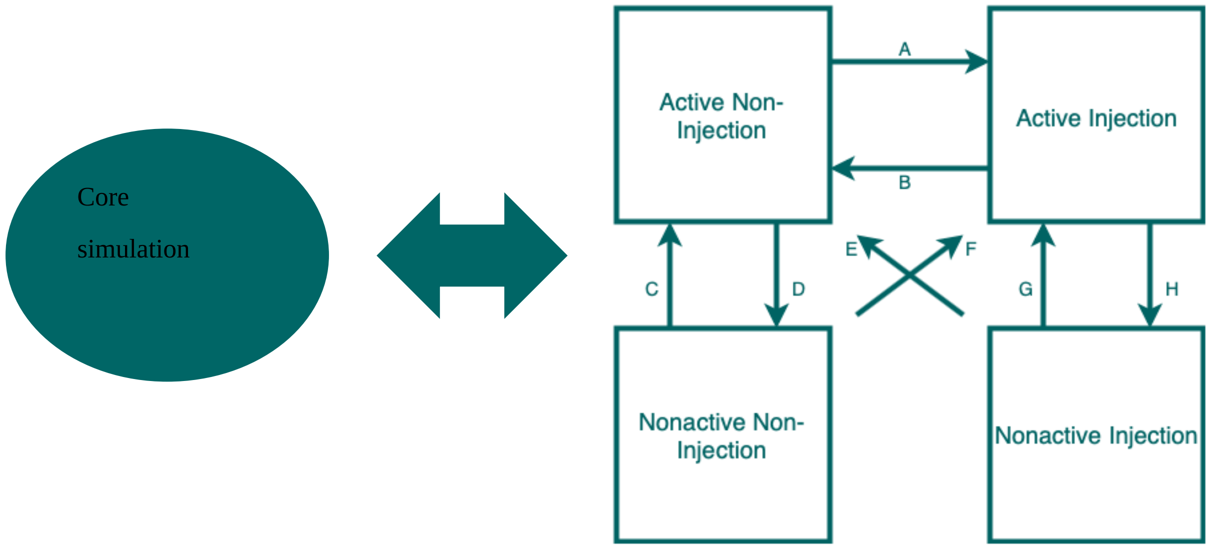

eFigure 2. Core Simulation

© 2025 Zwick H et al. *JAMA Network Open*

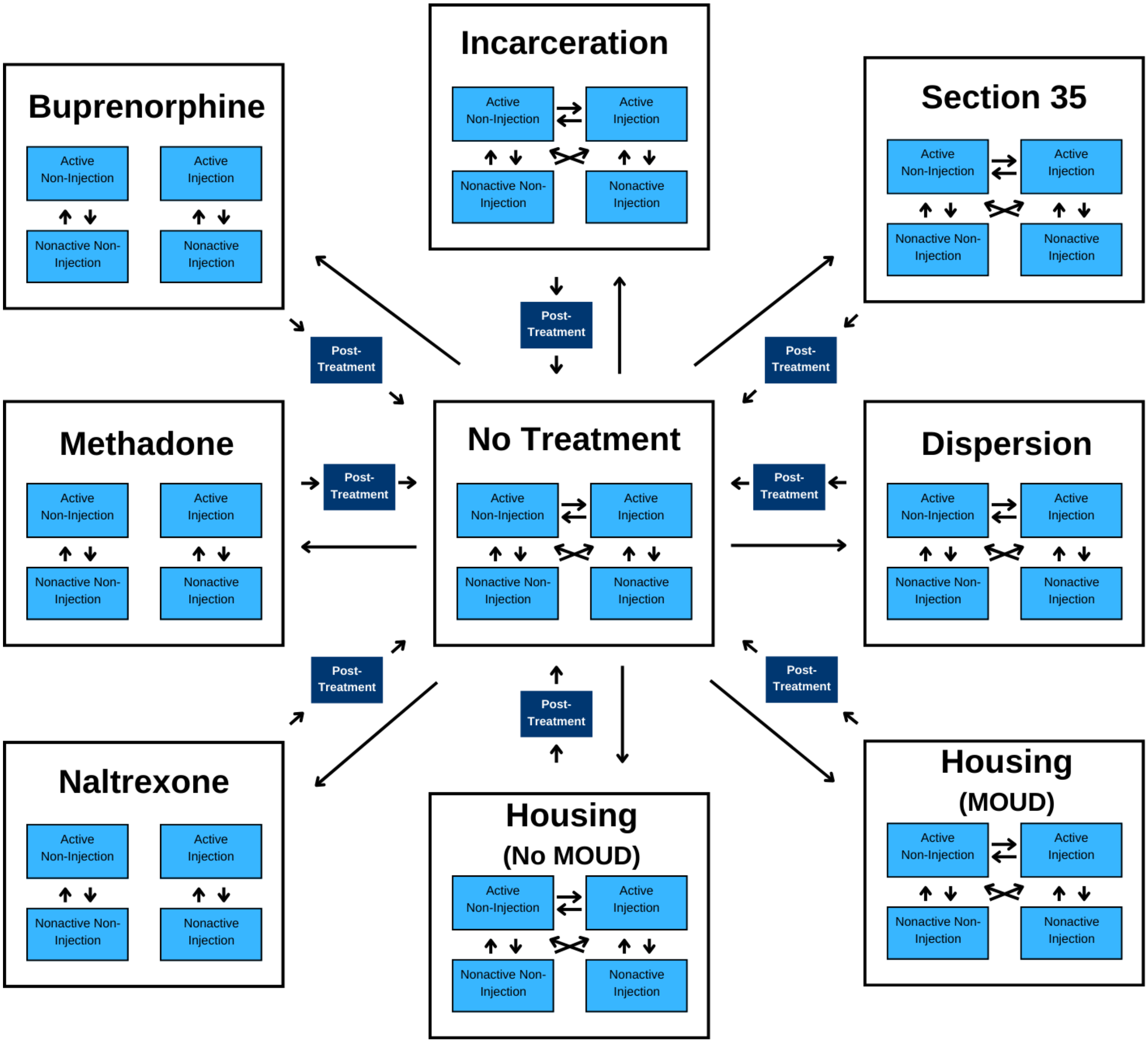

eFigure 2. RESPOND's care delivery module with project-specific strategies represented

© 2025 Zwick H et al. *JAMA Network Open*

C. Model Data and Methodology

C.1.1 Massachusetts Public Health Data Repository

The Massachusetts Public Health Data Repository (MA PHD) is a linked longitudinal records dataset that includes administrative records and billing claims from over 15 state agencies.[3] The spine of the database is the Massachusetts All Payers Claims database, which includes medical billing for all payers in the state. The database links person-level records from vital statistics, the Department of Corrections, Emergency Medical Services, and the Bureau of Substance Addiction Services, such that it is possible to construct longitudinal, person-level trajectories across various treatment episodes, admissions to the hospital, and overdose events. RESPOND uses the MA PHD dataset to estimate parameters such as OUD epidemiology in MA and rates of transition onto treatments assuming the status quo.

C.1.2 NIDA Clinical Trial Network Protocols 0051 (CTN)

The National Institute on Drug Abuse administers a large clinical trials network for evaluation of treatments for substance use disorders. The Clinical Trial Network (CTN) 0051 protocol was a head-to-head comparative effectiveness trial of sub-lingual buprenorphine and injectable naltrexone for individuals with OUD who were accessing acute opioid detoxification services. RESPOND uses urine toxicology data from the trial to estimate transitions between substance use states while taking buprenorphine or naltrexone, as well as health care utilization among patients with OUD.[4–6]

C.1.3 Medical Literature

In addition to the primary data sources listed above, RESPOND estimates many model parameters from the medical literature. The detailed explanation of model parameters below provides references to the relevant publications.

<u>C.2 Components</u>

C.2.1 Population Dynamics

C.2.1.1 Initial Cohort

To simulate the demography and OUD epidemiology in the underlying population, RESPOND requires the initial cohort to be specified as follows: 1) age and sex distributions of people with OUD, 2) proportion of people beginning in each drug-use state, and 3) proportion of people within each treatment episode.

Structural Assumptions:

- No population begins the simulation in a post-treatment block.
- RESPOND does not characterize the population by race or ethnicity.

Methodological Notes:

Table 1 presents the key parameters related to cohort initialization.

First, we took the estimates of the population of OUD between 2013 and 2015 from a age, sex, and county stratified capture-recapture analyses using the Massachusetts Public Health Data Warehouse (MA PHD).[7] The capture-recapture approach provides a method to estimate the total population with high-risk opioid use in a given calendar year, including those who have not been identified as being an opioid user and do not appear in medical claims or prevalence surveys. The previous work provides estimates for year 2012, however, as it has been noted in the paper, the data sources used before 2013 were not complete. Hence, we used data in the years with more comprehensive data sources to predict the data in 2012 which has fewer data sources.

Second, we obtained the age and sex stratified counts for alive OUD population at the end of each year by subtracting death with opioid overdose involved from the estimated total size.

Then, we applied a Negative Binomial regression on the alive counts to predict the counts in the end of 2012, with age, sex and calendar years being the covariates. An interaction of Age groups and sex was applied in the model. We treated this predicted count as the alive population size in the end of year 2012 and assumed that it constructs the initial cohort beginning in year 2013. To further identify the number of OUD in no treatment, as well as in treatment, at the beginning in year 2013, we identified OUD as we did in the capture-recapture and then counted the number of OUD in treatment (N_0) in January 2013. The remaining population size is called "the number of OUD in no treatment"(T_0).

eTable 1. Initializing Cohort Parameters

Parameter	Value	Method	Years	Stratification	Time Varying	Source
**Population size, n**					Yes	MA PHD analysis update to Barocas et al. [7]
**Population of high-risk opioid use**						
Total ($\hat{N}_{OUD,t}$)		Capture recapture	2013-2015	Age (3 groups*) & Sex	Yes	MA PHD analysis update to Barocas et al. [7]
**By age-group**		Observed	2012-2015	Age (18 groups**) & Sex	Yes	US Census 2010
**Proportion with injection drug use**	25.09%	Observed	2013	Age (3 groups*) & Sex	No	NSDUH
**Proportion non-actively using**	9%	Estimated			No	CDC [8] Cedarbaum et al [9]

Abbreviations:
- MA DPH: Massachusetts Department of Public Health data
- NSDUH: National Survey on Drug Use and Health

* 3 age groups: 10 – 24, 25 – 44, 45 – 99
** 18 age groups: 5-year age-groups from 10-99

© 2025 Zwick H et al. *JAMA Network Open*

C.2.1.2 Aging

RESPOND simulates discrete time steps (rather than continuous time) and categorical age groups or “brackets” over the lifetime. The user can define the bounds of age groups to match the structure of the underlying population. Aging occurs as the population progresses to the next age group after a number of cycles that is determined by the size of the age brackets. The model employs a half-cycle correction and aging occurs in discrete steps, namely only at multiples of the age group size.

Structural Assumptions:

- The entire population of the last age bracket (95 to 100-year-olds) is removed from the simulation at each aging cycle and replaced by the population from the previous age bracket.

C.2.1.3 Entering Cohort (New OUD Arrivals)

RESPOND can simulate either an open cohort (meaning that new individuals arrive to the population over the course of the simulation), or a closed cohort. Here we consider a **closed** cohort simulation of OUD population in state of Massachusetts. This means that there are no new entries to the population.

C.2.2 Natural History of OUD

RESPOND simulates opioid use as a series of transitions through four opioid use health states: 1) Non-Active and 2) Active non-injection use, as well as 3) Non-Active and 4) Active injection use (*Figure 1*). Throughout the simulation, there is a multi-directional movement between OUD states.

Transitions between drug use compartments impact four important outcomes: 1) risk of overdose, 2) risk of death from competing causes, 3) health care utilization (cost), and 4) quality of life.

The primary sources of data for substance use transitions are studies from the medical literature.

Structural Assumptions:

- OUD is a remitting and relapsing process over a lifetime. There is no health state of OUD cure or permanent recovery.
- Transitions between OUD health states are not time updated.

Methodological Notes:

eTable 2 presents the key parameters related to OUD transitions for no treatment.

- All Confidence Intervals (CIs) are 95%, namely calculated at α=5% level of significance.
- CIs for proportions p_E, p_F, p_B, p_H, and p_D are calculated using the normal approximation to binomial proportions.
- CIs for rates R_A, R_C, and R_D are provided from the manuscript and calculated assuming Poisson distribution.

© 2025 Zwick H et al. *JAMA Network Open*

- Weekly rates and proportions, calculated from the respective overall estimates, are converted to weekly transition probabilities as indicated in the "Method" column in **eTable 2**.

eTable 2. No-Treatment and Post-Treatment: OUD Transition Parameters

Parameter	Description	Value	Method	Source
**No Treatment (ρ_N)**				
**R_A**	Rate of active non-injection to active injection	4.6 per 100 PY<br>(3.0 , 6.6)		
**P_A**	Probability of active non-injection to active injection	0.000884<br>(0.000577 , 0.001268)	Calculated from R_A:<br>P_A= 1-exp{R_A/52}	
**R_C**	Rate of non-active non-injection to active non-injection	16 per 100 PY		Neaigus, A., et al. [10]
**R_G**	Rate of non-active injection to active injection	(12.0 , 20.5)		
**P_C**	Probability of non-active non-injection to active non-injection	0.00307	Calculated from Rate(R_C):	
**P_G**	Probability of non-active injection to active injection	(0.00230 , 0.00393)	P= 1-exp{R_C/52}	
**p_B**	Proportion of active injection to active non-injection	0.34		
**P_B**	Probability of active injection to active non-injection	0.00067<br>(0.00054 , 0.0008)	Calculated from p_B:<br>1-exp(x)<br>where<br>x = ln(1-p_B)/(12*52)	
**p_E**	Proportion of non-active injection to active non-injection	0.13		Shah, N.G., et al. [11]
**p_F**	Proportion of non-active non-injection to active injection			
**P_E**	Probability of non-active injection to active non-injection		Calculated from p_E:	
**P_F**	Probability of non-active non-injection to active injection	0.000223<br>(0.000115 , 0.00034)	1-exp(x)<br>where<br>x = ln(1-p_E)/(12*52)	
**p_D**	Proportion of active non-injection to non-active non-injection	0.03		
**p_H**	Proportion of active injection to non-active injection	(0.0175 , 0.0425)		
**P_D**	Probability of active non-injection to non-active non-injection	0.00058<br>(0.00032 , 0.00085)	Calculated from p_D:<br>1-exp(x)	Nosyk, B., et al. [12]
**P_H**	Probability of active injection to non-active injection		where<br>x = ln(1-p_D)/(52)	
**Post-Treatment (ρ_P)**				
**P_A, P_B, P_D, P_E, P_F, P_H,**	Same estimates with no-treatment.			
**p^*_C**	Proportion of non-active non-injection to active non-injection	0.65	CIs are calculated using the normal	Bailey et al. [13]

© 2025 Zwick H et al. *JAMA Network Open*

**p^*_G**	Proportion of non-active injection to active injection		approximation to binomial proportions.	
**P_C**	Probability of non-active non-injection to active non-injection	0.2308	Calculated from p:<br>1-exp(x)<br>where<br>x = ln(1-p)/4	
**P_G**	Probability of non-active injection to active injection			

- p^*_C and p^*_G indicate the percentage of people relapsed within a month of discharge (after inpatient detoxification).
- The denominators for calculating weekly probabilities depend on whether the respective available proportion or rate estimates are yearly or monthly.

C.2.3 Care Delivery

RESPOND models OUD while engaged with treatment using the same 4-state opioid use simulation that it uses to model OUD without treatment. The 4-state OUD simulation is embedded within all treatment episodes (blocks), such that individuals may both remain engaged with treatment, but also experience periods of drug use relapse. Each treatment type has its own bi-directional transition probabilities between active and non-active use. The net movement between active and non-active use while engaged with treatment favors movement to non-active use over time.

RESPOND simulates treatment using the following parameters:

1. Probability of movement onto treatment from no treatment
2. Treatment initiation effect – the probability of ceasing active opioid use immediately after initiating treatment
3. Bi-directional movements between active and non-active opioid use while engaged with treatment
4. Probability of loss to follow-up

The population that is lost to follow-up (disengages from care) must pass through a “post-treatment period” before rejoining the simulation of OUD. The post-treatment period is a four-week time, immediately following discontinuation of a treatment, during which the risk of relapse to drug use is high, as is the risk of overdose. Population that survives the post-treatment period transitions back to the simulation of OUD without treatment.

C.2.3.1 Movement From No-Treatment to Treatment Episodes

Structural Assumptions:

- Only population in active opioid use states seeks OUD treatment. Population that is not currently using opioids does not seek treatment.

The main source of data to inform the probability of transition from no treatment to a treatment episode is the MA PHD.

Methodological Notes:

Let λ_{NT} denotes the weekly transition rates from no-treatment to treatment. Then, weekly transition probability from no-treatment to treatment is calculated from λ_{NT} as:

$$\widehat{\mathbf{P}}_{\mathbf{NoTrt \rightarrow Trt.T}} = 1\text{- exp } \{-\lambda_{NT}\} \qquad (1)$$

© 2025 Zwick H et al. *JAMA Network Open*

Where $\lambda_{NT} = \frac{\widehat{N}_{\mathbf{Obs,NoTrt \rightarrow Trt\,.T}}}{\widehat{N}_{\mathbf{Total,NoTrt \rightarrow Trt\,.T}}} \times \frac{1}{4}$.

Here $\widehat{N}_{\text{Obs,NoTrt} \rightarrow \text{Trt.T}}$: the observed number of people with OUD who transitioned from no-treatment to treatment T in January 2013

$\widehat{N}_{\text{Total,NoTrt} \rightarrow \text{Trt.T}}$: the total number of people with OUD "*at risk*" of transitioning from no-treatment to treatment T in January 2013

The weekly transition probability $\widehat{P}_{\text{NoTrt} \rightarrow \text{Trt.T}}$ is estimated using data from the MA PHD repository, and is stratified by age (16 groups: 5-year age-groups from 10-85, and >85years old), sex, and treatment (T= Detox, Mmt, Ntx, and Bup) (**eTable3**).[3]

eTable 3. Weekly Transition Probabilities From No-Treatment to Treatment

Age	Sex	Transition to Treatment			
		**Detox***	**Methadone**	**Naltrexone**	**Buprenorphine**
		$\widehat{\mathbf{P}}_{\mathbf{NoTrt \rightarrow Trt\,.T}} = 1 - e^{-\hat{\lambda}_{ND}}$	$\widehat{\mathbf{P}}_{\mathbf{NoTrt \rightarrow Trt\,.T}} = 1 - e^{-\lambda_{NM}}$	$\widehat{\mathbf{P}}_{\mathbf{NoTrt \rightarrow Trt\,.T}} = 1 - e^{-\lambda_{NX}}$	$\widehat{\mathbf{P}}_{\mathbf{NoTrt \rightarrow Trt\,.T}} = 1 - e^{-\lambda_{NB}}$
10-14	Male	0.0037	0.0037	0.0037	0.0037
10-14	Female	0.0064	0.0064	0.0064	0.0064
15-19	Male	0.0027	0.0004	0.0016	0.0023
15-19	Female	0.0027	0.0005	0.0016	0.0027
20-24	Male	0.0054	0.0009	0.0012	0.0051
20-24	Female	0.005	0.0016	0.001	0.0057
25-29	Male	0.0056	0.0016	0.0007	0.0062
25-29	Female	0.0042	0.0028	0.0007	0.0065
30-34	Male	0.0053	0.002	0.0006	0.0068
30-34	Female	0.0035	0.0027	0.0006	0.0071
35-39	Male	0.0049	0.0018	0.0007	0.0068
35-39	Female	0.003	0.0021	0.0008	0.0066
40-44	Male	0.0046	0.0017	0.0006	0.0061
40-44	Female	0.0027	0.0021	0.0005	0.0057
45-49	Male	0.0038	0.0017	0.0005	0.0052
45-49	Female	0.0021	0.0015	0.0005	0.0051
50-54	Male	0.0032	0.0012	0.0005	0.0051
50-54	Female	0.0016	0.0013	0.0005	0.0048
55-59	Male	0.0025	0.0013	0.0004	0.0048
55-59	Female	0.001	0.0013	0.0007	0.0044
60-64	Male	0.0018	0.0018	0.0004	0.0044
60-64	Female	0.0012	0.0009	0.0003	0.004
65-69	Male	0.0014	0.0014	0.0005	0.0037
65-69	Female	0.0011	0.0005	0.0005	0.0038

© 2025 Zwick H et al. *JAMA Network Open*

70-74	Male	0.001	0.001	0.001	0.0031
70-74	Female	0.0009	0.0009	0.0009	0.0045
75-79	Male	0.002	0.002	0.002	0.0039
75-79	Female	0.0013	0.0013	0.0013	0.0053
80-84	Male	0.0062	0.0031	0.0031	0.0062
80-84	Female	0.0026	0.0013	0.0013	0.0065
85-99	Male	0.0035	0.0035	0.0035	0.0069
85-99	Female	0.0011	0.0011	0.0011	0.0057
* Detox transition probabilities listed here needed to be calibrated to match observed (from MA PHD) detox admissions each year from 2013-2015. We calibrated time varying multipliers $\boldsymbol{\eta}(\boldsymbol{t})$on approximate rates $\tilde{\lambda}_{ND}$ to obtain accurate estimates for detox transition rates $\lambda_{ND}(t)$ where $t = 2013, 2014, 2015$. In other words, $\lambda_{ND}(t)$ are calculated as $\lambda_{ND}(t) = \tilde{\lambda}_{ND} \times \boldsymbol{\eta}(\boldsymbol{t})$.					

C.2.3.2 Treatment Initiation Effect

When population begins a treatment for OUD, for example out-patient buprenorphine, a portion of the population immediately transitions from active to non-active use. Following that initial “treatment initiation effect” there is bidirectional movement between active and nonactive use states, even while engaged with treatment. The main source of data for the treatment initiation effect and for substance use transitions while engaged with buprenorphine, naltrexone, or methadone is the NIDA CTN urine toxicology data. The CTN trials collected routine periodic urine toxicology from all participants. While the published clinical trials results censored participants at the first relapse to drug use (the primary outcome of that trial), the trials continued to collect data from patients who experienced a relapse, such that the database includes longitudinal urine toxicology from patients who relapsed to active use, as well as some who remitted back to non-active use over the course of the trial. We analyzed those data in an “as treated” manner, such that RESPOND estimates realistic movements between active and non-active drug use states among people who are taking a medication. Note that relapsing to active drug use is not the same thing as loss to follow-up from treatment (see below).

Methodological Notes:

Upon entering treatment, a proportion of the population immediately transitions from active to non-active opioid use. This proportion $\hat{p}_{Init\_Act□NonAct}$ is stratified by treatment episode as follows:

- buprenorphine (Bup): 0.74, based on the proportion of observed negative (non-active) urine samples at week 1
- naltrexone (Ntx): 0.90, based on the proportion of observed negative urine samples at week 5
- methadone (Mmt): 0.57, based on the proportion of observed negative urine samples at week 5

We assume a binomial distribution and we use the Wald’s method to calculate 95% CIs for the proportion $\hat{p}_{Act□NonAct}$ representing the block initiation effect.

eTable 4. Block Initiation Effects Parameters: Weekly Transition Probabilities Modeling Movement Between OUD States When Movement Between Treatment States Occurs

**Initial OUD state**	**Transition to**			
	**Treatment (γ_T)**			**Post-treatment* (γ_P)**
	**Bup**	**Ntx**	**Mmt**	

© 2025 Zwick H et al. *JAMA Network Open*

**Active non-injection**	0.257(0.204 , 0.309)	0.103(0.058 , 0.148)	0.433(0.403 , 0.462)	1
**Active injection**	0.257(0.204 , 0.309)	0.103(0.058 , 0.148)	0.433(0.403 , 0.462)	1
**Non-active non-injection**	N/A**	N/A**	N/A**	N/A ***
**Non-active injection**	N/A**	N/A**	N/A**	N/A ***

* These estimates are the same for Bup, Ntx, Mmt, and detox.

** There is no block initiation effect for population that is not currently using opioids, because only population that is currently using opioids seeks care in the model.

*** Calibration parameter.

C.2.3.3 Transitions Between Active and Non-Active Opioid Use While Engaged with Treatment

We estimated Weekly OUD transition probabilities $\hat{p}_{Trt\_Act \square NonAct}$ using Multi-State Models.[14] We fit separate models for each treatment: buprenorphine (Bup), naltrexone (Ntx), and methadone (Mmt), using data from the National Institute of Drug Abuse Clinical Trials Network (NIDA CTN).[4–6]

Structural Assumptions:

- Population engaged with treatment may move between active and non-active opioid use, but the population engaged with treatment does not change the route of administration of their opioid use. In other words, population that entered treatment using non-injection opioids will not escalate to injection drug use while still engaged with treatment (Core Simulation within OUD treatment episodes (blocks) – (**eFigure**).

Transition probabilities between active and non-active states are the same for both injection and non-injection drug use. This structural assumption is confirmed by the MSM estimates for buprenorphine and methadone models, in which route was included as a model covariate, but was not a significant predictor of transition rates

Methodological Notes:

- Each MSM includes age and sex as covariates.
- Age is included as a continuous covariate in the MSM model, thus allowing estimation of the transition probabilities for age bins in which data are not available. We consider five 5 age groups: 10–19, 20–24, 25–34, 35–49, and 50–99 years old.
- OUD transition for Buprenorphine and Methadone: We keep all the weekly MSM estimates of OUD transition probabilities except week 1, which is considered as block initiation.
- OUD transition for Naltrexone: We delete the estimates for the first 4 weeks due to the inaccurate results from detoxification. Week 5 is also excluded from the analysis, as it is considered as block initiation.
- Transition probabilities from non-active to active use are defined as: $\hat{p}_{Trt\_NonAct \square Act} = 1 \square \hat{p}_{Trt\_Act \square NonAct}$

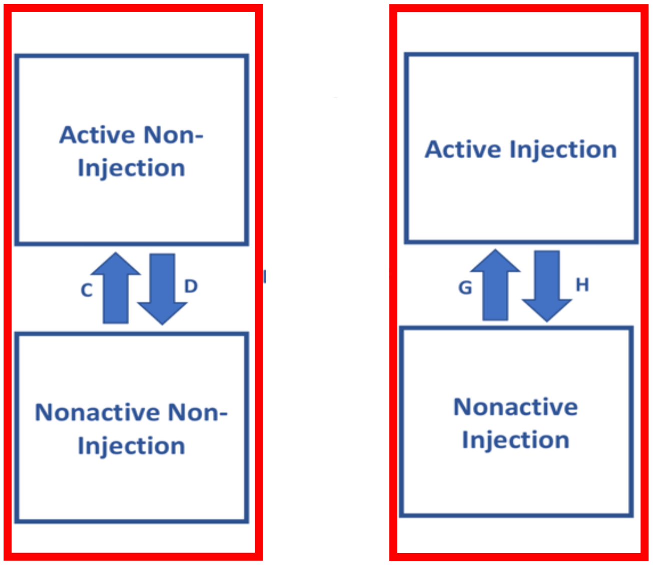

eFigure 3. Core Simulation Within OUD Treatment Episodes (blocks)

eTable 5. Transition Probabilities Between Active and Non-Active Opioid Use While Engaged with Treatment

Age	Sex	Initial OUD status*	Treatment		
			Buprenorphine	Naltrexone	Methadone
10-19	Male	Active	0.750	0.847	0.669
10-19	Female	Active	0.746	0.711	0.692
10-19	Male	Nonactive	0.156	0.107	0.112
10-19	Female	Nonactive	0.118	0.130	0.089
20-24	Male	Active	0.738	0.832	0.678
20-24	Female	Active	0.735	0.687	0.700
20-24	Male	Nonactive	0.148	0.099	0.120
20-24	Female	Nonactive	0.112	0.119	0.095
25-39	Male	Active	0.723	0.814	0.688
25-39	Female	Active	0.721	0.656	0.710
25-39	Male	Nonactive	0.139	0.090	0.128
25-39	Female	Nonactive	0.105	0.108	0.102
40-54	Male	Active	0.686	0.763	0.713
40-54	Female	Active	0.683	0.573	0.732
40-54	Male	Nonactive	0.119	0.072	0.152
40-54	Female	Nonactive	0.090	0.083	0.121
55-99	Male	Active	0.628	0.673	0.746
55-99	Female	Active	0.626	0.443	0.762
55-99	Male	Nonactive	0.096	0.051	0.192
55-99	Female	Nonactive	0.072	0.056	0.154

© 2025 Zwick H et al. *JAMA Network Open*

* Probabilities listed here are transition probabilities to "Active" state. For ex: - when initial OUD status is "Active", probabilities listed here are probabilities of staying in the "Active" state.

C.2.3.4 Probability of Loss to Follow-Up

In every time step, the population that is engaged with treatment faces a risk of disengaging from care and being lost to follow-up. Loss to follow-up differs from relapse to active drug use while remaining engaged with opioid treatment. The population that disengages with care and is lost to follow-up enters the "post-treatment state," during which time they have a high rate of relapse to active use and a high rate of overdose among active users. The post-treatment block represents the period immediately following discontinuation of a medication or release from an abstinence-based setting (acute drug detoxification center, residential drug treatment, or jail), when opioid tolerance is low, and the risk of overdose is higher than that of a person who never initiated treatment.

The main source of data for estimating the probability of loss to follow-up is Market Scan, a large insurance claims database containing millions of individuals who have commercial insurance coverage. As a randomized controlled trial, the CTN data cannot provide estimates of retention in care or loss to follow-up in the real world. We have previously published rates of loss to follow-up from buprenorphine and naltrexone treatment.[15] We therefore turn to Market Scan, which is nationally representative and reflects real-world practice in the U.S.

Structural Assumptions:

- In RESPOND, the only way to transition into a post-treatment episode is from a corresponding treatment episode.
- The "No Treatment" block does not have a post-treatment episode.
- RESPOND also considers the probability of immediate relapse to active opioid use upon being lost to follow-up from treatment:

Methodological Notes:

The weekly transition probability from treatment to post-treatment is calculated as:

$$\widehat{\mathbf{P}}_{\mathbf{Trt} \rightarrow \mathbf{Post-Trt\,.T}} = 1 - \exp\left(-\lambda_{TP}\right) \qquad (3)$$

where

$\lambda_{TP} = \frac{-log\{1-(1-p_T)\}}{t}$ and p_T: the retention probability for treatment T reported for the study time-period t in literature.

eeTable 6**6** presents estimates of the weekly transition probabilities $\widehat{\mathrm{P}}_{\mathrm{Trt} \rightarrow \mathrm{Post-Trt\,.T}}$ based on data from Morgan et al., stratified by treatment.[15]

eTable 6. Weekly Transition Probabilities from Treatment *T* to Post-Treatment

**Treatment *T***	p_T	**Study time-period *t***	λ_{TP}	$\widehat{\mathrm{P}}_{\mathrm{Trt} \rightarrow \mathrm{Post-Trt\,.T}}$	**Source**
**Bup**	0.1760	52 weeks	0.0334	0.0328	Morgan et al. [15]
**Naltrexone**	0.0214	52 weeks	0.0739	0.0713	
**Methadone**	0.5240	20 weeks	0.0323	0.0318	Strain et al. [16]

© 2025 Zwick H et al. *JAMA Network Open*

C.2.4 Overdose

Every person who is actively using opioids faces the risk of overdose. The probability of overdose depends on age, sex, and route of drug use (injection vs. non-injection). The simulation has no memory of past overdose events and does not include an elevated risk of repeat overdose after experiencing a first overdose event.

Structural Assumptions:

- Experiencing overdose has no independent impact on current or future opioid use behaviors.
- Only the population that is in an active opioid use state faces the risk of overdose.
- The risk of overdose is different between no-treatment, treatment, and post-treatment episodes.
- The risk of overdose is lower while engaged in treatment compared to not-engaged, even among the population who are actively using drugs while engaged with treatment.

Methodological Notes:

Counts of overdose are a target for model calibration. **eeTable 7** provides the empirically observed overdose fatalities from MA PHD.

eTable 7. Empirically Observed Opioid Overdoses in MA

**Year**	**Age**	**Sex**	**Total number of people with opioid overdose**	**Number of fatal opioid overdose**	**Number of people with non-fatal overdose**	**Total overdoses**
2013	10-19	Male	88	7	86	89
2013	10-19	Female	77	1	76	77
2013	20-24	Male	542	50	507	547
2013	20-24	Female	346	18	337	350
2013	25-39	Male	1929	264	1746	1966
2013	25-39	Female	975	99	899	995
2013	40-54	Male	1147	240	966	1178
2013	40-54	Female	776	121	673	791
2013	55+	Male	705	70	647	713
2013	55+	Female	630	30	596	631
2014	10-19	Male	112	13	107	113
2014	10-19	Female	101	1	101	101
2014	20-24	Male	823	75	778	833
2014	20-24	Female	502	33	482	508
2014	25-39	Male	3174	421	2898	3268
2014	25-39	Female	1415	141	1313	1443
2014	40-54	Male	1607	314	1368	1643
2014	40-54	Female	941	143	816	961
2014	55+	Male	913	105	825	928

© 2025 Zwick H et al. *JAMA Network Open*

2014	55+	Female	754	48	704	756
2015	10-19	Male	114	16	109	116
2015	10-19	Female	95	3	93	96
2015	20-24	Male	800	97	742	827
2015	20-24	Female	465	37	446	478
2015	25-39	Male	3804	545	3411	3914
2015	25-39	Female	1650	157	1537	1686
2015	40-54	Male	1863	357	1594	1919
2015	40-54	Female	973	142	871	1002
2015	55+	Male	1025	150	913	1045
2015	55+	Female	821	58	774	828

C.2.4.1 Overall (non-block specific) overdose rates

Yearly rate $R_{OD,t}$ of overdose at time t for people not engaged in treatment is calculated as:

$R_{OD,t} = \frac{N_{OD,t}}{N_{OUD,t}+\frac{1}{2}\cdot N_{enter,t}}$ □ 1 PY (4)

for years t= 2013, 2014, 2015, assuming that each person contributes 1 person/year

where

$N_{OD,t}$: number overdose cases at time t

$N_{OUD,t}$: OUD cohort size at time t

$N_{enter,t}$: entering cohort size at time t

Therefore, weekly overall overdose rate from anywhere in the model $o(t) = {R_{OD,t}}/{52}$.

eeTable presents point estimates of the overdose rates by age group, sex, OUD type, and year.

eTable 8. Opioid Overdose Rates by Age, Sex, Year, and Type of OUD

**Age**	**Sex**	**OUD**	**Years***		
			**2013**	**2014**	**2015**
10-19	Male	Active_Noninjection	0.000112076	0.000409791	0.000141081
10-19	Male	Active_Injection	0.000674857	0.002467526	0.000849508
10-19	Female	Active_Noninjection	0.000162217	0.000458584	0.000417609
10-19	Female	Active_Injection	0.000976778	0.002761329	0.002514598
20-24	Male	Active_Noninjection	0.001380621	0.000974182	0.000804356
20-24	Male	Active_Injection	0.008313304	0.005865957	0.004843369
20-24	Female	Active_Noninjection	0.001557061	0.001189503	0.000777667
20-24	Female	Active_Injection	0.00937572	0.007162497	0.004682664

© 2025 Zwick H et al. *JAMA Network Open*

25-39	Male	Active_Noninjection	0.000468337	0.000755758	0.00072912
25-39	Male	Active_Injection	0.002820052	0.004550736	0.004390337
25-39	Female	Active_Noninjection	0.00037227	0.000421042	0.000529921
25-39	Female	Active_Injection	0.002241592	0.002535274	0.003190876
40-54	Male	Active_Noninjection	0.000310914	0.000406988	0.000629183
40-54	Male	Active_Injection	0.001872141	0.002450645	0.003788574
40-54	Female	Active_Noninjection	0.000304432	0.000306566	0.000557584
40-54	Female	Active_Injection	0.001833113	0.001845965	0.003357447
55-99	Male	Active_Noninjection	0.000192418	0.000282608	0.000389897
55-99	Male	Active_Injection	0.001158631	0.0017017	0.002347731
55-99	Female	Active_Noninjection	0.00020927	0.000261909	0.000126691
55-99	Female	Active_Injection	0.0012601	0.001577063	0.000762859
* Overdose rates presented are weekly overall fixed rates o(t) for each year.					

Note that RESPOND model simulations has weekly time cycles. Let $\nu_B(\mathrm{t})$ be the overdose rate in a specific block where $B = N, T$ or P for blocks no-treatment, treatment, and post-treatment. Then, weekly overdose probabilities $P_{OD,B,t}$ are calculated from the respective overdose rates as:

$$P_{OD,B,t} = 1 - e^{-\nu_B(\mathrm{t})} \qquad (5)$$

where $B = N, T$ or P.

C.2.4.2 No Treatment

Weekly overdose rates in no treatment $\nu_N(\mathrm{t})$ were calculated by applying a multiplier $\mathbf{m_N} \in \mathbb{R}^+$ on overall overdose rates $\mathrm{o(t)}$ as $\nu_N(\mathrm{t}) = \mathrm{o(t)} \times \mathbf{m_N}$. There were no data available to inform the rate multiplier $\mathbf{m_N}$. Therefore, we decided to calibrate overdose rate multiplier of no treatment block.

C.2.4.3 Overdose While on Treatment

The risk of overdose for people engaged in treatment, is derived by applying a multiplier parameter $\mathbf{m_T}$ to the respective no-treatment $\nu_N(t)$ estimates. i.e., the weekly overdose rate at time t for treatment T is:

$$\nu_T(\mathrm{t}) = \nu_N(t) \times \mathbf{m_T} \qquad (6)$$

where $\nu_N(\mathrm{t})$ is the no-treatment overdose rate at time t, and $\mathbf{m_T} \in (0, 1)$..

© 2025 Zwick H et al. *JAMA Network Open*

eTable 9. Multipliers of Overdose Rates by Treatment *T*

Treatment T*	m_T	95% CI	Source
**Buprenorphine**	0.405	[0.35 , 0.46]	Morgan et al. [17]
**Naltrexone**	0.864	[0.42, 1.31] injectable	
**Methadone**	0.752**	Non-parametric uncertainty distribution from bootstrapping of data	Morgan et al. [17]<br>Sordo et al. [18]
***** We assume no overdoses occur in the detox block. Therefore, overdose rate is zero.<br>** $\mathbf{m_{Meth}} = \mathbf{m_{Bup}} \times \frac{\textit{Meth rate}}{\textit{Bup rate}} = 0.405 \times \frac{2.6}{1.4}$			

C.2.4.4 Overdose During the Post-Treatment Period

During the post-treatment period, individuals face a risk of overdose higher than that of people who never initiated a treatment. Therefore, we model post-treatment overdose rates $\nu_P(\mathrm{t})$ with a multiplier $\mathbf{m_P}$ (greater than 1) applied on no-treatment overdose rates such that $\nu_P(\mathrm{t}) = \nu_N(\mathrm{t}) \times \mathbf{m_P}$. There were no data available to inform post-treatment overdose rate multiplier $\mathbf{m_P}$. Therefore, we decided to calibrate overdose rate multiplier of post-treatments.

C.2.5 Mortality

RESPOND simulates mortality through two independent mechanisms, fatal opioid overdose and non-overdose death.

C.2.5.1 Fatal Overdose

The population that experiences overdose then faces a probability of death conditional on having had an overdose. This conditional probability of death, given an opioid overdose, is generalizable to all overdose cases and is therefore not stratified by age, sex, or OUD status. The population that survives an overdose does not change substance use as a result of the overdose. The probability of death conditional on having experienced an overdose is a time updated variable, reflecting changes to drug supply over time. Adjusting the conditional probability of overdose death provides a mechanism to reflect the growing penetration of fentanyl in local drug supplies, which is a major dynamic underlying mounting overdose deaths in the US.

The probability $\mathbf{f(t)}$ of fatal overdose at year t is calculated as:

$$\mathbf{f(t)} = \frac{\mathrm{N_{FOD,t}}}{\mathrm{N_{OD,t}}} \qquad (7)$$

where $\mathrm{N_{FOD,t}}$ is the total number of fatal overdoses, and $\mathrm{N_{OD,t}}$ is the total number of all-type overdoses.

© 2025 Zwick H et al. *JAMA Network Open*

eTable 10. Probabilities of fatal overdose

Year t	$\mathbf{f(t)}$
2013	0.1248 (0.1161, 0.1338)*
2014	0.1251 (0.1179, 0.1324)*
2015	0.1346 (0.1275, 0.1417)*
* Empirically calculated 95% CI using the Poisson distribution assumption for both counts in numerator and denominator of Eq. **Error! Reference source not found.**)	

C.2.5.2 Competing risks of death (non-overdose mortality)

Competing risks mortality includes deaths from conditions such as infectious endocarditis and sepsis, as well as medical comorbidities that accrue over a lifetime. The general approach to estimating competing risks of death is to apply standardized mortality ratios (SMRs) reflecting elevated mortality among drug users to age-sex stratified actuarial lifetables for the U.S.

eError! Reference source not found.**1Error! Reference source not found.** presents weekly death probabilities P_{NOD} calculated from lifetables in National Vital Statistic System after excluding overdoses. (National Center for Health Statistics)

eTable 21. Overdose excluded weekly death probabilities derived from lifetables

**Age**	**Sex**	**Probability of Death**
10-14	Male	3.23e-06
10-14	Female	2.33e-06
15-19	Male	1.22e-05
15-19	Female	5.32e-06
20-24	Male	2.20e-05
20-24	Female	7.85e-06
25-29	Male	2.43e-05
25-29	Female	1.00e-05
30-34	Male	2.77e-05
30-34	Female	1.41e-05
35-39	Male	3.37e-05
35-39	Female	1.90e-05
40-44	Male	4.50e-05
40-44	Female	2.83e-05
45-49	Male	6.83e-05
45-49	Female	4.41e-05
50-54	Male	0.000113101
50-54	Female	7.06e-05

55-59	Male	0.000173492
55-59	Female	0.000105002
60-64	Male	0.000253182
60-64	Female	0.000149513
65-69	Male	0.000350328
65-69	Female	0.000223448
70-74	Male	0.000535512
70-74	Female	0.000363913
75-79	Male	0.00084698
75-79	Female	0.000601651
80-84	Male	0.001427294
80-84	Female	0.001055789
85-89	Male	0.002907422
85-89	Female	0.002393584
90-94	Male	0.002907422
90-94	Female	0.002393584
95-99	Male	0.002907422
95-99	Female	0.002393584

Weekly non-overdose death rates R_{NOD} are calculated as

$$R_{NOD} = \ln(1 - P_{NOD}) \times SMR \qquad (8)$$

where SMR is calculated as:

$$\text{SMR} = \frac{\text{N}_{\text{D\_other}}}{\text{R}_\text{D} \times \text{N}_{\text{OUD}}} \qquad (9)$$

where N_{D_other} is the number of observed deaths not due to opioid overdose, R_D is the census death rate, and N_{OUD} is the size of the OUD population based on chapter 55 estimation.

We construct CIs around the SMRs estimates assuming that the N_{D_other} follows a Poisson distribution and using the normal approximation (**eTable 12**).

eTable 12. Standardized Mortality Rates (SMRs)

**Sex**	**OUD type**	**SMRs**<br>**(95% CIs)**
Male	Active - Non-injection	1.79(1.58 , 2.00)

© 2025 Zwick H et al. *JAMA Network Open*

Male	Active - Injection	4.41(3.84 , 4.98)
Male	Non-active – Non-injection	1.83(1.15 , 2.50)
Male	Non-active - Injection	4.59(2.71 , 6.46)
Female	Active – Non-injection	2.31(1.99 , 2.63)
Female	Active - Injection	5.67(4.81 , 6.53)
Female	Non-active – Non-injection	2.30(1.29 , 3.31)
Female	Non-active - Injection	5.62(2.87 , 8.37)

Finally, we convert weekly non-overdose death rates to probabilities as 1 □ $e^{-R_{NOD}}$.

C.2.5.3 Summary of the combined impact of medications for OUD on all-cause mortality in RESPOND

Medications for OUD (MOUD) have two independent effects on mortality that combine to provide synergies in the simulation:

- The population that is engaged with MOUD treatment experiences a net movement toward non-active drug use. Because there is no risk of overdose while not using drugs, MOUD tend to decrease the rate of overdose in the population. In addition, movement out of active drug use states reduces exposure to the high standardized mortality ratios (SMRs) of active drug use and thereby reduce non-overdose mortality as well.
- Among those who are actively using drugs when taking an MOUD, the MOUD has an independent effect on overdose risk, such that even those who are using have lower risk of death than those who are using drugs while not engaged with MOUD treatment

C.2.6 Parameter Summary

eTable 13. Model Parameters

**Parameter**	**Baseline Value**	**Range Evaluated**	**Source**
Population Demographics and Epidemiology			
Male, proportion of total people at baseline	0.58	N/A	[3,39]
Mean age, years<br>(Standard Deviation)	48<br>(17)	N/A	[3]
Proportion on Medication for Opioid Use Disorder at baseline	0.20	N/A	[3]
Injection drug use, proportion of total people at baseline	0.25	N/A	[40(p20)]
Active drug use, proportion of total people at baseline	0.91	N/A	[8,9]
Standardized mortality rate (SMR) for injection drug use	8.09	7.3 – 8.9	[3,41]
Standardized Mortality Rate for non-injection drug use	3.27	2.9 – 3.6	[3,41]
Transition to MOUD[‡] treatment and Housing, monthly rate per 1000 people			

© 2025 Zwick H et al. *JAMA Network Open*

Buprenorphine	11.6	10.4 – 12.7	[3]
Methadone	3.7	3.3 – 4.0	[3]
Naltrexone (injectable)	2.4	2.2 – 2.6	[3]
Housing (MOUD)	17.6	15.9 – 19.3	Summation of those who would move to MOUD
Housing (No MOUD)	17.1	15.4 – 18.8	[42]
Housing (MOUD) from Housing (No MOUD)	21.65	0 – 43.3	[43]
Retained on MOUD treatment and in Housing, proportion at 6 months			
Buprenorphine	0.42	0.19 – 0.90	[3]
Methadone	0.43	0.20 – 0.93	[3]
Naltrexone (injectable)	0.15	0.07- 0.32	[3]
Housing	0.98	0.44 - 1	[32,37,44–47]
Retention in Section 35 (days)			
Section 35	20	1 - 90	[48]
Overdose, monthly rate per 1,000 people*			
No Treatment	7.5	6.5 – 8.5	[3]
Buprenorphine	3.0	2.5 – 3.6	[3]
Methadone	5.3	3.0 – 7.0	[3]
Naltrexone (injectable)	5.4	2.6 – 7.3	[3]
Corrections	3.8	3.3 – 4.3	Half of “No Treatment” [49]
Section 35 (Mandatory Treatment)	0	0 – 4.3	Expert Opinion
Dispersion	7.5	6.5 – 8.5	Same as “No Treatment”
Housing (MOUD)	5.3	3.0 – 7.0	Same as Methadone
Housing (No MOUD)	7.5	6.5 – 8.5	Same as “No Treatment”
Fatal Overdoses, Proportion of total overdoses			
All settings	0.28	0.25 – 0.31	[3,50]
Pharmaceutical Cost			
Buprenorphine (weekly, $)	49	N/A	[51]
Methadone (weekly, $)	4	N/A	[51]
Naltrexone (weekly, $)	303	N/A	[51]
Housing (MOUD), (weekly, $)	4	N/A	Same as Methadone

© 2025 Zwick H et al. *JAMA Network Open*

Treatment Utilization Cost			
Buprenorphine<br>(weekly, $)	65	N/A	Expert opinion
Methadone<br>(weekly, $)	122	N/A	[52]
Naltrexone (injectable)<br>(weekly, $)	24	N/A	Expert opinion
Corrections<br>(weekly, $)	840	N/A	[53]
Section 35<br>(weekly, $)	1,477	N/A	[38]
Housing (MOUD)<br>(weekly, $)	293 + 122	N/A	Cost of Methadone [52]<br>+<br>Cost of Housing [32,54–56]
Housing (No MOUD)<br>(weekly, $)	293	N/A	Cost of Housing [32,54–56]
Overdose Cost			
Fatal overdose cost<br>($)	886	N/A	[3,25,27]
Non-fatal overdose cost<br>($)	4,557	N/A	[3,25,27]
Healthcare Utilization Cost, Weekly			
Buprenorphine<br>(weekly average, $)	226	N/A	[5,19,57]
Methadone<br>(weekly average, $)	226	N/A	[5,19,57]
Naltrexone<br>(weekly average, $)	289	N/A	[5,19,57]
Housing (MOUD)<br>(weekly average, $)	155	N/A	[32,35,37]
Housing (No MOUD)<br>(weekly average, $)	155	N/A	[32,35,37]
Cost of a Sweep			
Total<br>($ per person)	275	156.15 – 393.74	-
Component 1: Police<br>($ per person)	126	82 - 171	[28,29]
Component 2: Garbage and Sanitation<br>($ per person)	149	74 - 223	[29,30]

* A Probabilistic Sensitivity Analysis (PSA) was run on this parameter, meaning that in all 1,000 simulations, this parameter value was sampled from a distribution. All other parameters were ranged by hand, generally tested on one simulation.

ᵼ MOUD = Medications for Opioid Use Disorder

© 2025 Zwick H et al. *JAMA Network Open*

D. Empirical Calibration

D.1.1 Calibration Summary

We used an empirical calibration approach, using a Latin-Hypercube Sampling Design, to search the multidimensional parameter space and find parameter values for which simulations from the calibrated RESPOND model are very close to observed historical MA fatal overdoses, the size of the population using opioids (identified by the Massachusetts Public Health Data Warehouse and inflated using a capture-recapture analysis[7]), and counts of admission to medically supervised opioid withdrawal (detox) services.

The algorithm accepted proposed parameter values with respective model outputs within pre-specified uncertainty ranges for each target (OUD count, detox admissions, and fatal overdoses). The range for the 2015 total OUD count target is based on a published 95% confidence interval (CI) estimate.[7] The detox admissions and fatal overdose uncertainty ranges were defined to be within 10% of the observed count. Additionally, we rejected sampled parameters that did not result in an increasing trend in OUD counts from 2013 to 2015.

The result of the calibration exercise was a set of approximately 6,000 parameter vectors that provided simulations for historical trends of the outcomes of interest within the prespecified uncertainty ranges. We implemented internal and external validation methods and face validity tests to evaluate the model performance including a series of robustness checks, extreme value analysis and comparison to data sources and published studies not used in the calibration process.

D.1.2 Calibration Parameters

eTable 14. List of Calibration Parameters of RESPOND

Model parameter		Stratification and time dependency	Data source	Calibration parameter?
1. Entering cohort counts<br><br>(3 different parameters for three years)		➢ Yearly time varying (i.e., considered fixed per cycle within a single year)	Barocas, 2018 [7]	No
2. Entering cohort demographic proportions<br><br>(10 parameters each year = 30 altogether)		➢ Age-gender stratified<br>➢ Yearly time varying (i.e., considered fixed per cycle within a single year)	NSDUH dataset from 2013-2015	No
3. Block transition probabilities	1. Transitions from no-treatment to treatment blocks: Bup, Ntx, Meth and Detox (i.e., treatment initiation probabilities)<br><br>(16*2*4=128 parameters)	➢ Age-gender stratified<br>➢ Not stratified by route: injection vs non-injection for active users and non-active users are not allowed to transition to treatment blocks<br>➢ Stratified by treatment blocks<br>➢ No time variability	Chapter 55	No
	2. Transition probabilities for transitions from a treatment block to its post-treatment episode for Bup, Ntx, and Meth. For detox this probability is fixed at 1.<br><br>(3*2=6 parameters)	➢ Not age-gender stratified<br>➢ Stratified only by the active/non-active OUD status not by route<br>➢ Stratified by treatment blocks<br>➢ No time variability	Morgan, 2018 [15]	No
	3. Transition probabilities for transitions from post-treatment episodes to no-treatment block<br><br>(1 parameter)	➢ Fixed value at 1/4	Expert opinion	No

© 2025 Zwick H et al. *JAMA Network Open*

4. Block initiation effects (probability of keeping the same OUD state when transitioning to a different block)<br><br>(3*2+4*2)=14 parameters		➢ Stratified by destination blocks (For Detox, fixed at zero, and no initiation effect in no-treatment block)<br>➢ When transitioning to MOUD blocks, non-active users keep the same OUD state with probability 1. When transitioning to post-treatment episodes, active users keep the same OUD state with probability 1<br>➢ Does not differ by the initial block that someone is transitioning from	Treatment initiation effects are estimated from CTN data.<br><br>Bailey, 2013 [13]	Yes
5. OUD transition probabilities	1. OUD transitions in no-treatment block and post-treatment episodes<br><br>80 parameters altogether	➢ Age stratified but not stratified by gender<br>➢ Stratified by all four OUD states (no transitions allowed between non-active states, from active non-injection to non-active injection, from active injection to non-active non-injection<br>➢ Not stratified by blocks for post-treatment episodes (i.e., the same values are used in all post-treatment episodes)	Several literature manuscripts.	Yes
	2. OUD transitions in treatment blocks: Bup, Ntx, and Meth (i.e., treatment efficacy). For Detox, we assume people keep their initial OUD status and therefore, fixed.<br><br>60 parameters	➢ Stratified by both age and gender<br>➢ Only stratified by active vs non-active OUD states without route<br>➢ Stratified by blocks and multi-state models are used to estimate the probabilities in each block of MOUDs	CTN data	No
6. All types overdose proportions	1. All types overdose proportions in no-treatment block and post-treatment episodes of Bup, Ntx, Meth and Detox<br><br>Altogether 60 parameters for three years only for no-treatment block + a multiplier.<br><br>For post-treatment episodes we have another 60 parameters	➢ Age-gender stratified<br>➢ Stratified by route: injection vs non-injection for active users<br>➢ Yearly time varying (i.e., considered fixed per cycle within a single year)<br>➢ Not stratified by blocks for post-treatment episodes (i.e., the same values are used in all post-treatment episodes)	Chapter 55	Yes
	2. All types overdose proportions in treatment blocks: Bup, Ntx, and Meth, and zero for Detox<br><br>3 multipliers for each treatment	➢ Calculated as a multiplier on the values of no-treatment blocks. Therefore, same stratification and time dependency as no-treatment block.	Several literature manuscripts	Yes
7. Fatal overdose proportions (Calculated condition on overdoses)<br><br>Altogether 3 parameters for three years		➢ Yearly time varying (i.e., considered fixed per cycle within a single year)	Chapter 55	No
8. Standard Mortality Ratios (SMR)<br><br>8 parameters		➢ Stratified by gender and OUD status<br>➢ Not stratified by block. (i.e., same parameter values are used in all blocks)	Barocas, 2018 [7]<br><br>MA PHD [3]	No

© 2025 Zwick H et al. *JAMA Network Open*

E. Costs

E.1.1 Healthcare Utilization Costs

Healthcare utilization costs are defined as all non-MOUD-related costs within the healthcare system. Cost data come from Murphy et al., 2018, which used NIDA CTN-0051 data to quantify healthcare utilization costs stratified by age, opioid use status, and treatment setting (including no treatment, buprenorphine, and naltrexone).[19] Methadone costs were set as equal to buprenorphine costs. Detox was set to $0. Corrections costs are included in the modified societal perspective and use the federal inmate daily cost of incarceration fee.[20]

E.1.2 Pharmaceutical Costs

Pharmaceutical costs are the costs of the medication itself (buprenorphine/methadone/naltrexone). These costs come from the Federal Supply Schedule[21] and are presented in **eTable 15**.

eTable 15. Pharmaceutical Costs

Medication	Weekly Cost
Buprenorphine	$48.71
Naltrexone	$302.58
Methadone	$4.31
Detox	0

E.1.3 Treatment Utilization Costs

Treatment Utilization costs are the cost of the regimen of provider visits and related medical testing necessary for engagement with an MOUD. These costs do not include medication costs. Expert opinion provided the procedures during a standard visit for buprenorphine and for naltrexone, which were then costed using Medicare data.[22,23] Methadone costs came from the NIDA website.[24] Detoxification costs came from the NIDA CTN-0051 trial.[19]

eTable 16. Cost of Naltrexone Treatment for Opioid Use Disorder

	Non-facility (Nonfac) total	Conversion factor	Cost-NonFac	Annual Cost-Nonfac	Weekly Cost-Nonfac
**Quarterly MD visit**					
**Visit New**					
Level 3 [CPT 99203]	3.05	36.0391	109.919255	54.96	
Level 4 [CPT 99204]	4.63	36.0391	166.861033	83.43	
**Visit Established**					
Level 3 [CPT 99213]	2.09	36.0391	75.321719	112.98	
Level 4 [CPT 99214]	3.06	36.0391	110.279646	165.42	
**Monthly Nurse visit** [CPT 99211]	0.64	36.0391	23.065024	184.52	
**Monthly depot injection** [CPT 96327]			16.94	203.28	
**Annual HIV test** [CPT 86701]			9.87	9.87	

**Annual Hepatitis A/B/C test** [CPT 86704, 86706, 86708, 86803, 87340]	66.41	66.41	
**Annual syphilis blood test** [CPT 86593]	4.89	4.89	
**Annual gonorrhea/ chlamydia test** [CPT 87491, 87591]	77.98	77.98	
**Monthly urine toxicology screen** [CPT 80306]	17.14	205.68	
	**Total Cost:**	1169.42	24.36296632

eTable 17. Cost of Buprenorphine Treatment for Opioid Use Disorder

	Non-facility total	Conversion factor	Cost-NonFac	Annual Cost-Nonfac	Weekly Cost-NonFac
**Monthly MD visit**					
**Visit New**					
Level 3 [CPT 99203]	3.05	36.0391	109.919255	54.96	
Level 4 [CPT 99204]	4.63	36.0391	166.861033	83.43	
**Visit Established**					
Level 3 [CPT 99213]	2.09	36.0391	75.321719	414.27	
Level 4 [CPT 99214]	3.06	36.0391	110.279646	606.54	
**Weekly Nurse visit** [CPT 99211]	0.64	36.0391	23.065024	922.60	
**Annual HIV test** [CPT 86701]			00009.87	9.87	
**Annual Hepatitis A/B/C test** [CPT 86704, 86706, 86708, 86803, 87340]			66.41	66.41	
**Annual syphilis blood test** [CPT 86593]			00004.89	4.89	
**Annual gonorrhea/ chlamydia test** [CPT 87491, 87591]			77.98	77.98	
**Weekly urine toxicology screen** [CPT code 80306]			00017.14	891.28	
			**Total Cost:**	3132.23	65.25476274

eTable 18. Cost of Methadone Treatment for Opioid Use Disorder

Annual Cost	Weekly Cost	Weekly cost minus medication	Source
6552	126	121.69	NIDA

E.1.4 Overdose Costs

Overdose costs are the cost of an overdose, including emergency room and inpatient treatment.

Non-fatal overdose costs: Of 2014 overdoses in RI, ~60% went to the ER only and 40% were then inpatient.[25] Cost for an ER stay was $908 in 2016 dollars, which is $962.48 in 2019 dollars.[19,26] Total costs (not charges) of all inpatient treatment for OUD was $13,799,794 (in 2019 dollars) which was approximately $9120 per person.[25] This means for ~60% of all overdoses, the cost was ED only and for 40% of overdoses the cost was inpatient costs. This gives an equation of 0.6*928.48 + 0.4*9120.82. In addition, we add cost of ambulance to everybody, which is an additional $331.10 (converted to 2019 dollars).[27]

eTable 19. Cost of Nonfatal Overdose

	Total Visits	%	Cost
ED Visits	2269	0.59994712	962.48
Hospital Visits	1513	0.40005288	9120.815572
Ambulance	All		331.1
**Total Cost:**	4557.35		

Fatal overdose costs: Using MA PHD data, in 2013-2015 Massachusetts had 29,167 all types of overdoses and 3,756 fatal overdoses. Some of the non-fatal overdoses would likely not have been observed, but this is difficult to account for. To attempt to do this, we used death percentages from Jiang et al to predict how many of the all-types overdose deaths would likely have happened in a hospital setting.[25] Of 29,167 deaths, we assume 60% (17,500) were observed in the ED and 40% (11,667) inpatient. Jiang et al had a 1.4% fatal OD rate in the ED and 6.2% fatal OD rate inpatient. Using our numbers, this means we could expect 53 fatal overdoses in the ED and 233 inpatient. This leaves 3,470 fatal overdoses unaccounted for (unobserved by the healthcare sector). So- of 3,756 fatal overdoses, 53 are assigned a cost of ED only, 233 are assigned cost of inpatient, and 3,470 are assigned cost of ambulance only (we assume all fatal overdoses, even those dead on ambulance arrival, would incur an ambulance cost) 26. With that in mind, fatal overdose cost comes to $885.97.

eTable 20. Cost of Fatal Overdose

		Counts	Deaths	Percent total fatal OD	Cost of fatal OD
Fatal OD (2013-2015)		3756	3756		
All type opioid OD (2013-2015)		29167			
	ER	17500.2	52.5006	0.013977796	962.48
	Inpatient	11666.8	233.336	0.062123536	9120.82
Unobserved fatal OD			3470.1634	0.923898669	331.1
**Total Cost:**					885.97

E.1.5 Intervention Costs

eTable 21. Cost of Interventions

Category	Sub-Category	Cost per person, USD	Sources
Police Sweep	Police Costs	126.41	Bedford, WGBH [28]<br>U.S. Department of Housing and Urban Development. [29]
	Sanitation/Cleaning Costs	148.54	The Denver Post [30]<br>U.S. Department of Housing and Urban Development. [29]
	Total Costs	274.95	
Housing, per week<br><br>(*same sources used to characterize housing)	Total Costs	293	Wood, 2018 [31]<br>Massachusetts Housing and Shelter Alliance. [32]<br>Basu, 2012. [33]<br>How to close the housing gap through strategic partnerships. [34]<br>Brown, 2015. [35]<br>Lim, 2018. [36]<br>Massachusetts Housing and Shelter Alliance. Permanent Supportive Housing: A Solution-Driven Model. [37]
Section 35, per week	Total Costs	1,477.23	WBUR [38]

© 2025 Zwick H et al. *JAMA Network Open*

F. Sensitivity Analyses

F.1.1 Sensitivity Analyses

The 6000 parameter vectors resulting from the Empirical Calibration represent the empirical multidimensional distribution of the model parameters (only the ones that we attempted to calibrate). The set of these vectors can be viewed as a non-parametric approximation of the true (but unknown) underlying multivariate distribution of the model parameters.

We randomly selected 1000 vectors from this empirical multivariate distribution, and we ran the model for each one of these vectors to account for the parameter uncertainty in the calibrated parameters. Therefore, the resulting uncertainty intervals (eTable23) of the simulated outcomes of interest also capture the parameter uncertainty of the RESPOND model. In that sense the analysis we performed for the respective results presented in table **eTable 23** is indeed a type of probabilistic sensitivity analysis (PSA) where we convey the uncertainty around the calibrated parameters using their respective multivariate empirical distribution resulting from the empirical calibration.

We took the mean of each outcome value to compare the strategies. To assess uncertainty, **eFigure 4** plots fatal overdoses across strategies for each parameter set, 1 – 1,000. The smoothed lines confirm that for any given parameter set, the ranking of the strategies when considering fatal overdoses remains the same. The difficulty with presenting typical uncertainty estimates is that the overlaps are not a true comparison of strategies, as they do not consider the parameter set. For example, parameter set 750 for the 'Housing' strategy crosses the smoothed 'Sweep' line, indicating that with some combination of parameters, the 'Housing' strategy results in more fatal overdoses (~7) than the average of the 'Sweep strategy (~6.75). However, at parameter set 750, we also see a large peak, indicating that when 'Housing' results in a higher number of fatal overdoses (~7), so does 'Sweep' (~7.75).

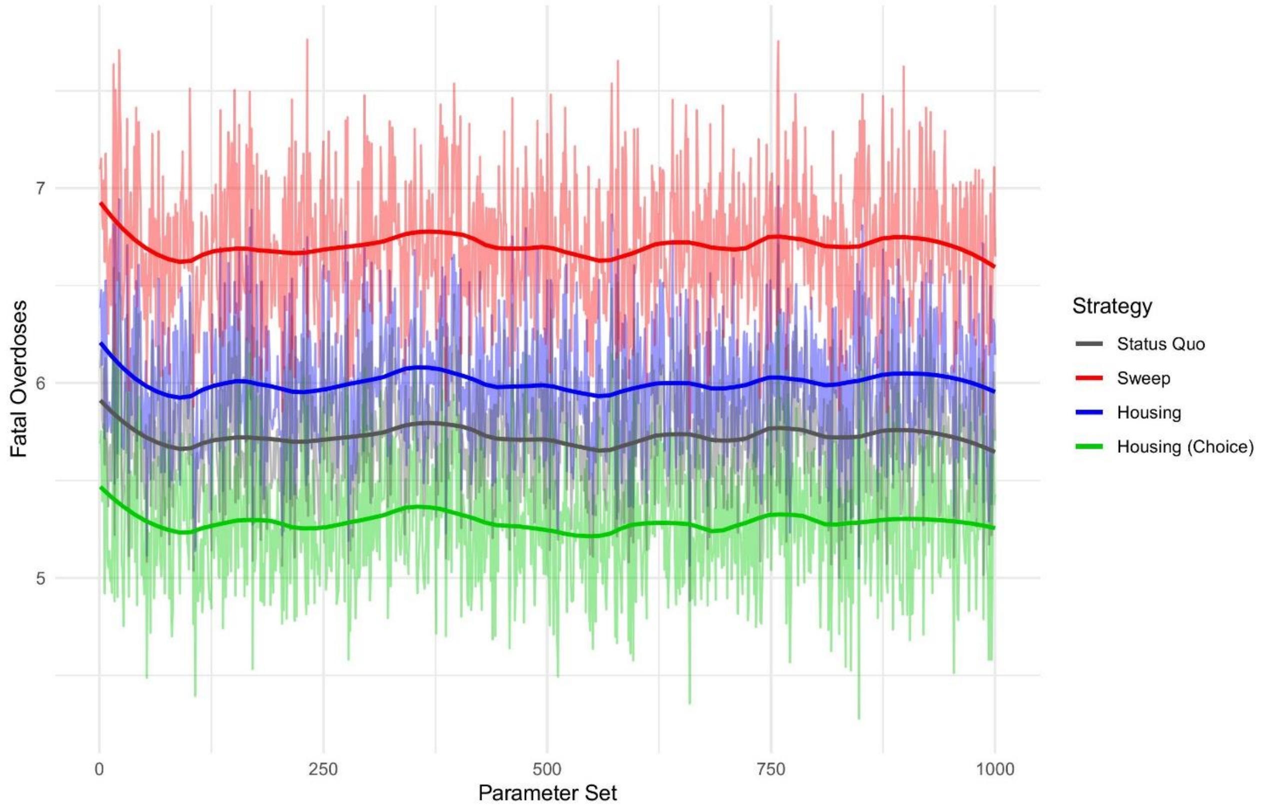

eFigure 4. Fatal Overdoses by Strategy and Parameter Set

© 2025 Zwick H et al. *JAMA Network Open*

Deterministic Sensitivity Analyses (DSAs) were conducted on further parameters that did not vary between parameter sets, including movement between intervention settings, retention probabilities, the fatal overdose proportion, and more. Many were conducted across all four strategies, though some were strategy specific.

Upper and lower bounds for PSAs and DSAs are listed in **eTable 22**. Bounds differ for various reasons, including the possibility of hitting the maximum bound and expert opinion.

eTable 22. Sensitivity analyses upper and lower bounds

Parameter	Strategies	Lower Bound	Upper Bound
**General**			
Opioid use transitions: to Active Use in all treatment states	SQ<br>SW<br>HM<br>HC	25th percentile of 1000 runs	75th percentile of 1000 runs
Movements onto MOUD	SQ<br>SW<br>HM<br>HC	Combined rate of movement to all MOUDs decreased by 10%	Combined rate of movement to all MOUDs increased by 10%
Overdose rates	SQ<br>SW<br>HM<br>HC	5th percentile of 1000 runs	95th percentile of 1000 runs
Fatal overdose proportion	SQ<br>SW<br>HM<br>HC	Decreased 10%	Increased 10%
SMR	SQ<br>SW<br>HM<br>HC	Decreased 10%	Increased 10%
MOUD and Housing Retention	SQ<br>SW<br>HM<br>HC	Decreased 3%	Increased 3%
MOUD proportions	SQ	Switched proportions of individuals on Buprenorphine and Methadone	
**Sweep-Specific**			
Movement to Section 35 at week 53	SW	0% movement to Section 35	20% movement to Section 35
MOUD engagement at week 53	SW	0%	Triple the default
Overdose probability in Section 35	SW	0%	Equal to that of Corrections
**Housing-Specific**			
Proportion moving from housing (no meds) to housing (meds)	HC	0%	Double the default
Movement to housing (meds) from No Treatment during week 53	HM	0%	15%

© 2025 Zwick H et al. *JAMA Network Open*

Overdose probability in housing	HM HC	Decreased 10%	Increased 10%

F.1.2 Sensitivity Analyses Output

DSAs are conducted on one input set, rather than the full 1,000, due to time and efficiency concerns. Therefore, results of original runs do not look the same as in the full results. Sensitivity analyses are intended to test relative changes, so it is sufficient to review the changes from one input (the same input file is used across all DSAs) and draw conclusions from the relative changes presented below. DSA results are reported in **eTable 23**.

eTable 23. Sensitivity Analysis Results

**General DSAs**					
	**Movement to MOUD**				
	**Fatal ODs**	**Total Fatalities**	**Cost**	**Weeks in Housing**	**Weeks in MOUD**
SQ Low	6.16	19.03	$6,624,702	0	2,805
SQ	6.08	18.95	$6,609,054	0	3,022
SQ High	6.00	18.86	$6,593,985	0	3,231
SW Low	7.15	20.00	$6,954,741	0	1,585
SW	7.10	19.94	$6,942,329	0	1,719
SW High	7.04	19.99	$6,930,240	0	1,850
HM Low	6.45	19.31	$7,214,919	2,818	2,818
HM	6.38	19.24	$7,262,395	3,054	3,054
HM High	6.32	19.18	$7,308,499	3,283	3,283
HC Low	5.77	18.63	$8,782,339	14,404	4,790
HC	5.69	18.55	$8,814,147	14,257	5,032
HC High	5.61	18.47	$8,844,808	14,115	5,266
	**Opioid Use Transitions**				
	**Fatal ODs**	**Total Fatalities**	**Cost**	**Weeks in Housing**	**Weeks in MOUD**
SQ Low	5.91	18.71	$6,569,369	0	2,980
SQ	6.08	18.95	$6,609,054	0	3,022
SQ High	6.26	19.12	$6,652,028	0	3,068

© 2025 Zwick H et al. *JAMA Network Open*

SW Low	6.91	19.69	$6,902,656	0	1,691
SW	7.10	19.94	$6,942,329	0	1,719
SW High	7.30	20.14	$6,985,309	0	1,751
HM Low	6.22	19.00	$7,223,216	3,045	3,045
HM	6.38	19.24	$7,262,395	3,054	3,054
HM High	6.57	19.42	$7,304,745	3,066	3,066
HC Low	5.53	18.32	$8,786,990	14,492	5,000
HC	5.69	18.55	$8,814,147	14,257	5,032
HC High	5.85	18.71	$8,844,577	14,441	5,070
	**Overdose Rate**				
	**Fatal ODs**	**Total Fatalities**	**Cost**	**Weeks in Housing**	**Weeks in MOUD**
SQ Low	4.97	17.89	$6,631,692	0	3,035
SQ	6.08	18.95	$6,609,054	0	3,022
SQ High	6.58	19.42	$6,498,952	0	3,016
SW Low	5.78	18.68	$6,969,446	0	1,729
SW	7.10	19.94	$6,942,329	0	1,719
SW High	7.76	20.58	$6,928,801	0	1,715
HM Low	5.15	18.06	$7,287,315	3,069	3,069
HM	6.38	19.24	$7,262,395	3,054	3,054
HM High	6.85	19.69	$7,251,492	3,050	3,050
HC Low	4.58	17.49	$8,844,901	14,568	5,057
HC	5.69	18.55	$8,814,147	14,257	5,032
HC High	5.95	18.79	$8,800,661	14,505	5,026
	**Fatal Overdose Rate**				
	**Fatal ODs**	**Total Fatalities**	**Cost**	**Weeks in Housing**	**Weeks in MOUD**
SQ Low	5.50	18.40	$6,630,765	0	3,028

© 2025 Zwick H et al. *JAMA Network Open*

SQ	6.08	18.95	$6,609,054	0	3,022
SQ High	6.65	19.49	$6,587,502	0	3,015
SW Low	6.43	19.30	$6,967,482	0	1,724
SW	7.10	19.94	$6,942,329	0	1,719
SW High	7.76	20.58	$6,918,302	0	1,715
HM Low	5.78	18.67	$7,285,719	3,061	3,061
HM	6.38	19.24	$7,262,395	3,054	3,054
HM High	6.98	19.81	$7,239,241	3,048	3,048
HC Low	5.15	18.04	$8,839,339	14,534	5,042
HC	5.69	18.55	$8,814,147	14,257	5,032
HC High	6.22	19.06	$8,789,129	14,485	5,022
	**SMR**				
	**Fatal ODs**	**Total Fatalities**	**Cost**	**Weeks in Housing**	**Weeks in MOUD**
SQ Low	6.10	18.04	$6,642,817	0	3,037
SQ	6.08	18.95	$6,609,054	0	3,022
SQ High	6.05	19.80	$6,576,317	0	3,008
SW Low	7.12	19.04	$6,866.992	0	1,729
SW	7.10	19.94	$6,832,349	0	1,719
SW High	7.06	20.80	$6,798,753	0	1,710
HM Low	6.41	18.34	$7,299,509	3,070	3,070
HM	6.38	19.24	$7,262,395	3,054	3,054
HM High	6.36	20.10	$7,226,412	3,039	3,039
HC Low	5.71	17.65	$8,859,428	14,575	5,058
HC	5.69	18.55	$8,814,147	14,257	5,032
HC High	5.66	19.41	$8,770,235	14,425	5,008
	**MOUD and Housing Retention**				

© 2025 Zwick H et al. *JAMA Network Open*

	Fatal ODs	Total Fatalities	Cost	Weeks in Housing	Weeks in MOUD
SQ Low	6.48	19.35	$6,644,128	0	1,735
SQ	6.08	18.95	$6,609,054	0	3,022
SQ High	4.45	17.32	$6,446,529	0	7,956
SW Low	7.25	20.10	$6,842,385	0	1,177
SW	7.10	19.94	$6,942,329<br>$6,832,349	0	1,719
SW High	6.68	19.52	$6,796,670	0	3,209
HM Low	6.74	19.60	$6,975,907	1,748	1,748
HM	6.38	19.24	$7,262,395	3,054	3,054
HM High	4.93	17.79	$8.344,709	8,065	8,065
HC<br>Low	6.43	19.30	$7,853,530	8,436	2,418
HC	5.69	18.55	$8,814,147	14,257	5,032
HC High	4.76	17.61	$9,329,376	15,943	8,452
	**MOUD Proportions**				
	**Fatal ODs**	**Total Fatalities**	**Cost**	**Weeks in Housing**	**Weeks in MOUD**
SQ	5.71<br>(5.15 – 6.33)	18.56<br>(18.03 – 19.15)	$6,582,981<br>($6,501,723 - $6,660,188)	0	2,990<br>(2,897 – 3,081)
SQ MOUD SWAP	5.74<br>(5.15 – 6.36)	18.58<br>(17.99 – 19.18)	$6,559,642<br>($6,482,522 - $6,637,827)	0	3,010<br>(2,919 – 3,102)
Delta	+0.53%	+0.11%	-0.35%	-	+0.67%
**Sweep-Specific DSAs**					
	**MOUD Retention During Sweep**				
	**Fatal ODs**	**Total Fatalities**	**Cost**	**Weeks in Housing**	**Weeks in MOUD**
SW – No Engagement	7.20	20.05	$6,949,347	0	1,474
SW - Default	7.10	19.94	$6,942,329	0	1,719
SW - Double	6.99	19.84	$6,935,310	0	1,965

© 2025 Zwick H et al. *JAMA Network Open*

SW - Triple	6.89	19.74	$6,928,292	0	2,210
	**Movement to Section 35 During the Sweep**				
	**Fatal ODs**	**Total Fatalities**	**Cost**	**Weeks in Housing**	**Weeks in MOUD**
SW – 0%	7.09	19.94	$6,746,032<br>$6,636,052	0	1,661
SW - Default	7.10	19.94	$6,942,329<br>$6,832,349	0	1,719
SW – 20%	7.10	19.95	$7,138,625<br>$7,028,645	0	1,778
	**Overdose Rate in Section 35**				
	**Fatal ODs**	**Total Fatalities**	**Cost**	**Weeks in Housing**	**Weeks in MOUD**
SW – No Section Overdoses	7.10	19.94	$6,942,329<br>$6,832,349	0	1,719
SW – Increased Overdoses	7.14	19.98	$6,941,624<br>$6,831,644	0	1,719
Delta	+0.56%	+0.20%	-0.01%	-	0%
**Housing-Specific DSAs**					
	**Uptake of MOUD once housed**				
	**Fatal ODs**	**Total Fatalities**	**Cost**	**Weeks in Housing**	**Weeks in MOUD**
HC – 0%	5.99	18.85	$8,677,330	14,495	3,692
HC - Default	5.69	18.55	$8,814,147	14,257	5,032
HC - Double	5.44	18.30	$8,928,694	14,502	6,156
	**Uptake of Housing with MOUD from No Treatment during the Sweep**				
	**Fatal ODs**	**Total Fatalities**	**Cost**	**Weeks in Housing**	**Weeks in MOUD**
SQ	6.08	18.95	$6,609,054	0	3,022
SW	7.10	19.94	$6,942,329<br>$6,832,349	0	1,719
HM – No Engagement	6.38	19.24	$7,262,395	3,054	3,054
HM – 5%	6.33	19.18	$7,340,793	3,396	3,396

© 2025 Zwick H et al. *JAMA Network Open*

HM – 10%	6.27	19.12	$7,419,190	3,737	3,737
HM – 15%	6.21	19.06	$7,497,588	4,079	4,079
	**Overdose Rate in Housing**				
	**Fatal ODs**	**Total Fatalities**	**Cost**	**Weeks in Housing**	**Weeks in MOUD**
SQ	6.08	18.95	$6,609,054	0	3,022
SW	7.10	19.94	$6,942,329<br>$6,832,349	0	1,719
HM - Low	6.35	19.20	$7,262,404	3,055	3,055
HM	6.38	19.24	$7,262,395	3,054	3,054
HM - High	6.42	19.28	$7,262,386	3,054	3,054
HC – Low	5.29	18.16	$8,810,336	14,509	5,035
HC	5.69	18.55	$8,814,147	14,257	5,032
HC - High	6.08	18.94	$8,808,829	14,489	5,030

One important dynamic the impact of housing strategies, discovered through sensitivity analyses, for was the rate of acceptance of housing. In HM, total deaths exceeded SQ when fewer than (25.0%) of individuals accepted MOUD to secure housing (**eFigure 5**).

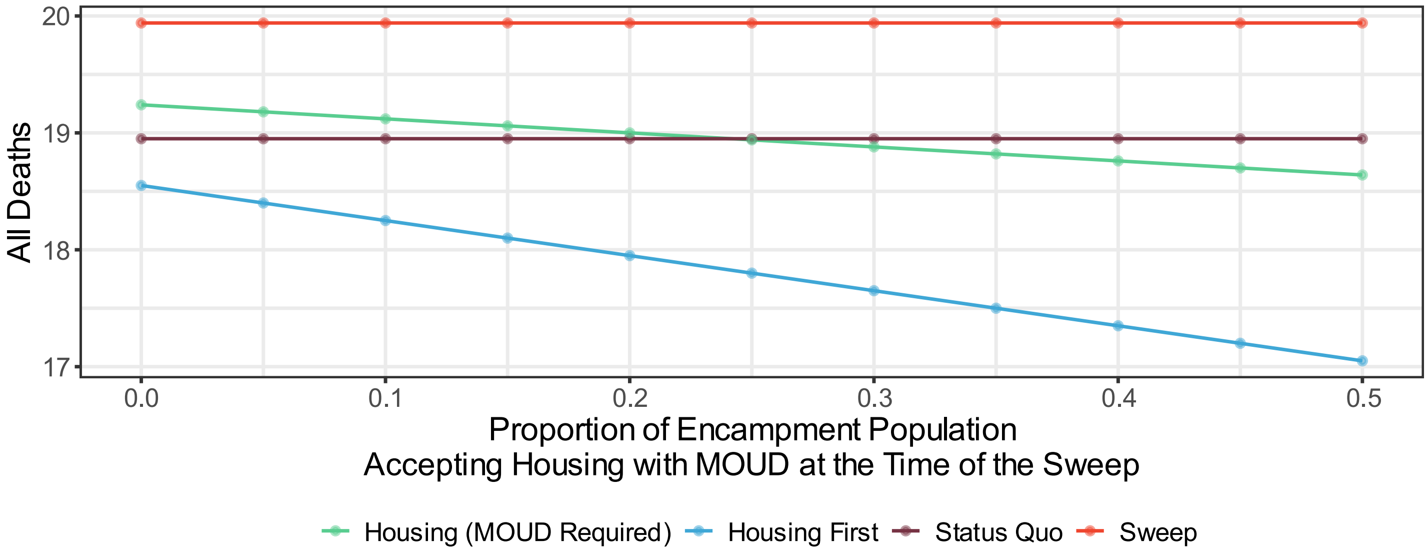

eFigure 5. Projected Deaths by Strategy and Proportion of Population Accepting Housing with MOUD at the Time of the Sweep

eReferences

1. Siebert U, Alagoz O, Bayoumi AM, et al. State-Transition Modeling: A Report of the ISPOR-SMDM Modeling Good Research Practices Task Force-3. *Value in Health*. 2012;15(6):812-820. doi:10.1016/j.jval.2012.06.014

2. Sonnenberg FA, Beck JR. Markov-Models in Medical Decision-Making - a Practical Guide. *Med Decis Making*. 1993;13(4):322-338. doi:10.1177/0272989x9301300409

3. Public Health Data Warehouse (PHD) | Mass.gov. Accessed January 11, 2023. https://www.mass.gov/public-health-data-warehouse-phd

4. Lee JD, Nunes EV, Mpa PN, et al. NIDA Clinical Trials Network CTN-0051, Extended-Release Naltrexone vs. Buprenorphine for Opioid Treatment (X:BOT): Study design and rationale. *Contemp Clin Trials*. 2016;50:253-264. doi:10.1016/j.cct.2016.08.004

5. Lee JD, Nunes EV Jr, Novo P, et al. Comparative effectiveness of extended-release naltrexone versus buprenorphine-naloxone for opioid relapse prevention (X:BOT): a multicentre, open-label, randomised controlled trial. *Lancet*. 2018;391(10118):309-318. doi:10.1016/S0140-6736(17)32812-X

6. Nunes EV, Krupitsky E, Ling W, et al. Treating Opioid Dependence With Injectable Extended-Release Naltrexone (XR-NTX): Who Will Respond? *Journal of Addiction Medicine*. 2015;9(3):238-243. doi:10.1097/ADM.0000000000000125

7. Barocas JA, White LF, Wang JN, et al. Estimated Prevalence of Opioid Use Disorder in Massachusetts, 2011-2015: A Capture-Recapture Analysis. *Am J Public Health*. 2018;108(12):1675-1681. doi:10.2105/Ajph.2018.304673

8. Centers for Disease Control and Prevention. HIV Infection Risk, Prevention, and Testing Behaviors Among Persons Who Inject Drugs. 2018. Accessed October 24, 2023. https://www.cdc.gov/hiv/pdf/library/reports/surveillance/cdc-hiv-surveillance-special-report-number-24.pdf

9. Cedarbaum ER, Banta-Green CJ. Health behaviors of young adult heroin injectors in the Seattle area. *Drug and Alcohol Dependence*. 2016;158:102-109. doi:10.1016/j.drugalcdep.2015.11.011

10. Neaigus A, Gyarmathy VA, Miller M, Frajzyngier VM, Friedman SR, Des Jarlais DC. Transitions to Injecting Drug Use Among Noninjecting Heroin Users: Social Network Influence and Individual Susceptibility. *JAIDS Journal of Acquired Immune Deficiency Syndromes*. 2006;41(4):493-503. doi:10.1097/01.qai.0000186391.49205.3b

11. Shah NG, Galai N, Celentano DD, Vlahov D, Strathdee SA. Longitudinal predictors of injection cessation and subsequent relapse among a cohort of injection drug users in Baltimore, MD, 1988-2000. *Drug Alcohol Depend*. 2006;83(2):147-156. doi:10.1016/j.drugalcdep.2005.11.007

12. Nosyk B, Li L, Evans E, et al. Characterizing longitudinal health state transitions among heroin, cocaine, and methamphetamine users. *Drug Alcohol Depend*. 2014;140:69-77. doi:10.1016/j.drugalcdep.2014.03.029

13. Bailey GL, Herman DS, Stein MD. Perceived relapse risk and desire for medication assisted treatment among persons seeking inpatient opiate detoxification. *J Subst Abuse Treat*. 2013;45(3):302-305. doi:10.1016/j.jsat.2013.04.002

14. Jackson C. Multi-State Models for Panel Data: The msm Package for R. *2011*. 2011;38(8):28. doi:10.18637/jss.v038.i08

15. Morgan JR, Schackman BR, Leff JA, Linas BP, Walley AY. Injectable naltrexone, oral naltrexone, and buprenorphine utilization and discontinuation among individuals treated for opioid use disorder in a United States commercially insured population. *J Subst Abuse Treat*. 2018;85:90-96. doi:10.1016/j.jsat.2017.07.001

16. Strain EC, Bigelow GE, Liebson IA, Stitzer ML. Moderate- vs High-Dose Methadone in the Treatment of Opioid Dependence: A Randomized Trial. *JAMA*. 1999;281(11):1000. doi:10.1001/jama.281.11.1000

17. Morgan JR, Schackman BR, Weinstein ZM, Walley AY, Linas BP. Overdose following initiation of naltrexone and buprenorphine medication treatment for opioid use disorder in a United States commercially insured cohort. *Drug and Alcohol Dependence*. 2019;200:34-39. doi:10.1016/j.drugalcdep.2019.02.031

© 2025 Zwick H et al. *JAMA Network Open*

18. Sordo L, Barrio G, Bravo MJ, et al. Mortality risk during and after opioid substitution treatment: systematic review and meta-analysis of cohort studies. *BMJ*. 2017;357:j1550. doi:10.1136/bmj.j1550

19. Murphy SM, McCollister KE, Leff JA, et al. Cost-effectiveness of buprenorphine–naloxone versus extended-release naltrexone to prevent opioid relapse. *Annals of Internal Medicine*. 2019;170(2):90-98. doi:10.7326/M18-0227

20. Annual Determination of Average Cost of Incarceration Fee (COIF). Federal Register. September 1, 2021. Accessed October 17, 2024. https://www.federalregister.gov/documents/2021/09/01/2021-18800/annual-determination-of-average-cost-of-incarceration-fee-coif

21. Logistics O of P Acquisition and. VA.gov | Veterans Affairs. Accessed October 17, 2024. https://www.va.gov/opal/nac/fss/pharmPrices.asp

22. Physician Fee Schedule | CMS. Accessed October 17, 2024. https://www.cms.gov/medicare/payment/fee-schedules/physician

23. Clinical Laboratory Fee Schedule | CMS. Accessed October 17, 2024. https://www.cms.gov/medicare/payment/fee-schedules/clinical-laboratory-fee-schedule-clfs

24. Abuse NI on D. How much does opioid treatment cost? | National Institute on Drug Abuse (NIDA). --. Accessed October 17, 2024. https://nida.nih.gov/publications/research-reports/medications-to-treat-opioid-addiction/how-much-does-opioid-treatment-cost

25. Jiang Y, McDonald JV, Koziol J, McCormick M, Viner-Brown S, Alexander-Scott N. Can Emergency Department, Hospital Discharge, and Death Data Be Used to Monitor Burden of Drug Overdose in Rhode Island? *J Public Health Manag Pract*. 2017;23(5):499-506. doi:10.1097/PHH.0000000000000514

26. Medical Expenditure Panel Survey Home. Accessed February 6, 2023. https://meps.ahrq.gov/mepsweb/index.jsp

27. Coffin PO, Sullivan SD. Cost-effectiveness of distributing naloxone to heroin users for lay overdose reversal. *Ann Intern Med*. 2013;158(1):1-9. doi:10.7326/0003-4819-158-1-201301010-00003

28. Bedford T. Boston Police run up millions in overtime at Mass. and Cass homeless encampment. News. September 26, 2022. Accessed July 7, 2023. https://www.wgbh.org/news/local-news/2022/09/26/boston-police-run-up-millions-in-overtime-at-mass-and-cass-homeless-encampment

29. U.S. Department of Housing and Urban Development. Exploring Homelessness Among People Living in Encampments and Associated Cost. https://huduser.gov/portal/sites/default/files/pdf/Exploring-Homelessness-Among-People.pdf

30. Denver's homeless sweeps cost hundreds of thousands of dollars, invoices show. The Denver Post. January 19, 2021. Accessed July 7, 2023. https://www.denverpost.com/2021/01/19/denver-homeless-sweeps-cost-2/

31. Wood L, Wood NJR, Vallesi S, Stafford A, Davies A, Cumming C. Hospital collaboration with a Housing First program to improve health outcomes for people experiencing homelessness. *Housing, Care and Support*. 2018;22(1):27-39. doi:10.1108/HCS-09-2018-0023

32. Massachusetts Housing and Shelter Alliance. Home_and_Healthy_for_Good.pdf. March 2009. Accessed July 7, 2023. https://shnny.org/uploads/Home_and_Healthy_for_Good.pdf

33. Basu A, Kee R, Buchanan D, Sadowski LS. Comparative Cost Analysis of Housing and Case Management Program for Chronically Ill Homeless Adults Compared to Usual Care. *Health Serv Res*. 2012;47(1 Pt 2):523-543. doi:10.1111/j.1475-6773.2011.01350.x

34. How to close the housing gap through strategic partnerships. Accessed October 2, 2023. https://www.advisory.com/topics/social-determinants-of-health/2017/09/how-to-close-the-housing-gap-through-strategic-partnerships

35. Brown RT, Miao Y, Mitchell SL, et al. Health Outcomes of Obtaining Housing Among Older Homeless Adults. *Am J Public Health*. 2015;105(7):1482-1488. doi:10.2105/AJPH.2014.302539

© 2025 Zwick H et al. *JAMA Network Open*

36. Lim S, Singh TP, Hall G, Walters S, Gould LH. Impact of a New York City Supportive Housing Program on Housing Stability and Preventable Health Care among Homeless Families. *Health Serv Res*. 2018;53(5):3437-3454. doi:10.1111/1475-6773.12849

37. Massachusetts Housing and Shelter Alliance. Permanent Supportive Housing: A Solution-Driven Model. December 2017. Accessed January 30, 2023. https://archives.lib.state.ma.us/bitstream/handle/2452/782511/ocn887735103-2017.pdf?sequence=1&isAllowed=y.

38. By The Numbers: Section 35 Civil Commitments. Accessed January 11, 2023. https://www.wbur.org/news/2019/04/10/civil-commitments-massachusetts-treatment

39. Rapid Assessment of Consumer Knowledge (RACK) - Opioid Policy Research Collaborative. Accessed October 4, 2023. https://heller.brandeis.edu/opioid-policy/community-resources/rack/index.html

40. Results from the 2015 National Survey on Drug Use and Health: Detailed Tables. Published online 2016. https://www.samhsa.gov/data/sites/default/files/NSDUH-DetTabs-2015/NSDUH-DetTabs-2015/NSDUH-DetTabs-2015.pdf

41. Roncarati JS, Baggett TP, O'Connell JJ, et al. Mortality Among Unsheltered Homeless Adults in Boston, Massachusetts, 2000-2009. *JAMA Intern Med*. 2018;178(9):1242-1248. doi:10.1001/jamainternmed.2018.2924

42. Workbook: Mass Cass Dashboard V2. Accessed January 2, 2023. https://dashboard.boston.gov/t/Guest_Access_Enabled/views/MassCassDashboardv2/HousingTab?%3Aembed=y&%3Aiid=4&%3AisGuestRedirectFromVizportal=y

43. Komaromy M, Stone A, Peterson A, Gott J, Koenig R, Taylor JL. Facilitating exit from encampments: combining low-barrier transitional housing with stabilizing treatment for substance related problems. *Addict Sci Clin Pract*. 2023;18(1):66. doi:10.1186/s13722-023-00420-y

44. Collins SE, Malone DK, Clifasefi SL. Housing Retention in Single-Site Housing First for Chronically Homeless Individuals With Severe Alcohol Problems. *Am J Public Health*. 2013;103(Suppl 2):S269-S274. doi:10.2105/AJPH.2013.301312

45. Davidson C, Neighbors C, Hall G, et al. Association of Housing First Implementation and Key Outcomes Among Homeless Persons With Problematic Substance Use. *PS*. 2014;65(11):1318-1324. doi:10.1176/appi.ps.201300195

46. Stefancic A, Tsemberis S. Housing First for Long-Term Shelter Dwellers with Psychiatric Disabilities in a Suburban County: A Four-Year Study of Housing Access and Retention. *J Primary Prevent*. 2007;28(3):265-279. doi:10.1007/s10935-007-0093-9

47. Bean KF, Shafer MS, Glennon M. The impact of housing first and peer support on people who are medically vulnerable and homeless. *Psychiatr Rehabil J*. 2013;36(1):48-50. doi:10.1037/h0094748

48. Massachusetts Department of Public Health. Section 35 Commission Treatment Statistics from BSAS Programs. February 28, 2019. Accessed January 2, 2023. https://www.mass.gov/files/documents/2019/03/04/DPH%20Section%2035%20Commission%202-28-2019.pdf

49. Chatterjee A, Weitz M, Savinkina A, et al. Estimated Costs and Outcomes Associated With Use and Nonuse of Medications for Opioid Use Disorder During Incarceration and at Release in Massachusetts. *JAMA Netw Open*. 2023;6(4):e237036. doi:10.1001/jamanetworkopen.2023.7036

50. O'Driscoll PT, McGough J, Hagan H, Thiede H, Critchlow C, Alexander ER. Predictors of accidental fatal drug overdose among a cohort of injection drug users. *Am J Public Health*. 2001;91(6):984-987.

51. Logistics O of P Acquisition and. Office of Procurement, Acquisition and Logistics (OPAL): Pharmaceutical Prices. Accessed October 24, 2023. https://www.va.gov/opal/nac/fss/pharmPrices.asp

52. National Institute on Drug Abuse. How much does opioid treatment cost? | National Institute on Drug Abuse (NIDA). --. Accessed November 2, 2023. https://nida.nih.gov/publications/research-reports/medications-to-treat-opioid-addiction/how-much-does-opioid-treatment-cost

53. Annual Determination of Average Cost of Incarceration Fee (COIF). Federal Register. September 22, 2023. Accessed February 6, 2025. https://www.federalregister.gov/documents/2023/09/22/2023-20585/annual-determination-of-average-cost-of-incarceration-fee-coif

54. Latimer EA, Rabouin D, Cao Z, et al. Cost-Effectiveness of Housing First With Assertive Community Treatment: Results From the Canadian At Home/Chez Soi Trial. *PS*. 2020;71(10):1020-1030. doi:10.1176/appi.ps.202000029

55. Aubry T, Bloch G, Brcic V, et al. Effectiveness of permanent supportive housing and income assistance interventions for homeless individuals in high-income countries: a systematic review. *The Lancet Public Health*. 2020;5(6):e342-e360. doi:10.1016/S2468-2667(20)30055-4

56. Jacob V, Chattopadhyay SK, Attipoe-Dorcoo S, et al. Permanent Supportive Housing With Housing First: Findings From a Community Guide Systematic Economic Review. *Am J Prev Med*. 2022;62(3):e188-e201. doi:10.1016/j.amepre.2021.08.009

57. McCollister KE, Leff JA, Yang X, et al. Cost of Pharmacotherapy for Opioid Use Disorders Following Inpatient Detoxification. *Am J Manag Care*. 2018;24(11):526-531.

© 2025 Zwick H et al. *JAMA Network Open*
