# Supplementary material for: Health and Economic Outcomes of Addressing Encampments of Individuals Using Opioids
Source: JAMA Netw Open. 2025 Jun 27;8(6):e2517095. doi: 10.1001/jamanetworkopen.2025.17095 (PMC12205401; doi:10.1001/jamanetworkopen.2025.17095)
Supplement: Supplement 2. — Data Sharing Statement [file jamanetwopen-e2517095-s002.pdf]

Data Sharing Statement

Zwick. Health and Economic Outcomes of Addressing Encampments of Individuals Using Opioids. *JAMA Netw Open*. Published June 25, 2025.
doi:10.1001/jamanetworkopen.2025.17095

Data
Data available: Yes
Data types: Data (not involving human participants)
How to access data: The data and code will be made available on Github: https://github.com/SyndemicsLab
When available: With publication

Supporting Documents
Document types: Statistical/analytic code
How to access documents: The data and code will be made available on Github: https://github.com/SyndemicsLab
When available: With publication

Additional Information
Who can access the data: The data will be publicly available.
Types of analyses: The data will be publicly available for any appropriate purpose, specifically simulation and health economics research.
Mechanisms of data availability: The data will be publicly available online, without investigator support necessary.
